# Molecular Dynamics Simulation of Nanostructured Grazynes for Water Desalination

**DOI:** 10.1021/acsanm.6c01675

**Published:** 2026-06-29

**Authors:** Adrià Calzada, Francesc Viñes, Pablo Gamallo

**Affiliations:** Departament de Ciència de Materials i Química Física & Institut de Química Teòrica i Computacional (IQTCUB), 16724 Universitat de Barcelona, c/Martí i Franquès 1-11, Barcelona 08028, Spain

**Keywords:** grazynes, desalination, water transport, salt rejection, membranes

## Abstract

The search for selective desalination membranes is a topic of great importance for producing freshwater. Here, the potential use of grazynes — 2D C-based porous membranes that combine graphene stripes linked by acetylenic linkages — as desalination membranes has been explored using molecular dynamics simulations. Five different grazynes with pore sizes between 1 and 21 Å^2^ were considered under pressures of 150 to 210 MPa. Two of these grazynes proved to be completely impermeable, while the other three — [1],[2]{2}, [1],[2]{3}, and [1],[3]{2} — demonstrated a clear water flow that increased almost linearly with pressure. Among these, [1],[3]{2} showed the highest water flow due to its larger effective pore size, which led to the passage of Na^+^ and Cl^–^ traces. The [1],[2]{2}-grazyne proved to be the best-balanced membrane between water flow and salt rejection, achieving even perfect salt rejection in the high-pressure range. When compared to other C-based membranes, grazynes achieved competitive results, reaching permeabilities of *ca.* 15 L·cm^–2^·day^–1^·MPa^–1^ under conditions of perfect salt rejection. Even at the highest pressure where salt ion permeation was observed, the resulting permeate salinity remained in a range associated with poor taste by the World Health Organization (WHO) while still being potentially suitable for irrigating many crops according to the Food and Agriculture Organization of the United Nations (FAO) salinity guidelines. These results position grazynes as candidates for desalination membranes and open pathways for further investigation.

## Graphical abstract

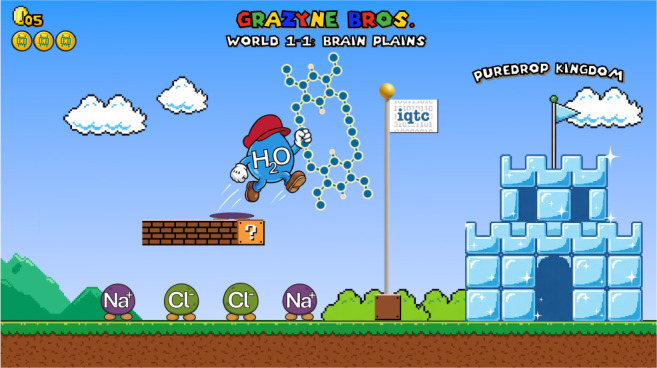

## 1 Introduction

The increasing global demand for freshwater is one of the nowadays largest problems, accentuated by the continuous population growth, the industrialization processes, and climatic change. This has intensified the necessity of having desalination technologies capable of working in an efficient yet sustainable way. Hitherto, freshwater deposits have failed to duly supply both population and industry with the needed water, prompting mankind to turn its attention into the oceans. The oceanic masses and seas represent *ca*. 97% of the global water, and are, by far, the first source of said resource. However, despite all the efforts made, it has not been able to harness this almost endless water (H_2_O) reservoir without a high energy consumption and high capital costs. It is in this context that the reverse osmosis (RO) process, based on semipermeable membranes, is considered as the most energy-efficient desalination process.^1^ However, prior to the moment when RO became the main technology, several methods and techniques were considered. The first methods were based on evaporation and condensation processes, including membrane distillation^2,3^ and thermal vapor compression (TVC) technologies,^4^ among others.^5^ The high energy demand of these techniques encouraged the search for alternative methods, such as filtration technologies. These are processes where membranes allow molecules to pass through them in different ways depending on their nature. These processes include technologies like forward osmosis (FO) and nanofiltration (NF).^6^ Despite all this, the most important process belonging to this family is RO, a membrane-based filtration process where dissolved solutes and impurities are removed from water by the application of pressure, driving water through a semipermeable membrane. This process operates against the natural osmotic gradient, allowing only water molecules to pass while rejecting larger particles such as Na^+^, Cl^–^, SO_4_^2–^, and Mg^2+^, among others.

Within the RO field, polyamide thin film composite (PA-TFC) membranes have been the market leaders since their production due to their excellent properties for treating saline water.^7^ However, even though these membranes may seem ideal for their use, much work still needs to be done on them, as they continue to suffer from severe fouling^8^ and sensitivity to oxidative agents.^9^ For these reasons, scientists are still searching for new materials — membranes — that can improve the existing ones and further optimize the desalination process. In this regard, in the past few years, and thanks to the discovery of graphene,^10^ carbon-based membranes have gained momentum in the field of molecular separation.^11,12^ These materials have attracted attention due to their exceptional mechanical strength, chemical stability, and tunable pore structures. Notable examples include nanoporous graphene, where pore chemistry has been shown to influence both water molecule transport efficiency and salt rejection.^13^ Additionally, other studies demonstrated that pore size is crucial to effectively separate water from its impurities.^14,15^ Other Carbon allotropes that have been studied include graphene oxide^16^ (GO) and carbon nanotubes (CNTs).^17^ Graphynes^18^ have also been studied as potential sieving or desalination membranes,^19^ achieving remarkable results with high water permeability and even reaching 100% salt rejection in some cases.^20^

In the field of molecular separation using C-based membranes, another promising family of materials is grazynes.^21^ These are single-layer structures that combine C atoms with both *sp* and *sp* ^2^ hybridizations, where graphene stripes are linked through acetylenic linkages. From an electronic perspective, grazynes demonstrate an exclusive way of transporting electrical current across their acetylenic connections.^22^ Moreover, grazynes present a high degree of tunability that allows one to make structural modifications for specific applications, either by creating vacancies or enlarging the acetylenic linkages. In the last years, some studies have explored the use of grazynes as filtration membranes of gas mixtures for technological applications, including biogas upgrading, demonstrating clear advantages over traditional scrubbing materials,^23^ and potential candidates for air purification, particularly for separating gas mixtures such as N_2_/CO_2_ and N_2_/O_2_,^24,25^ and even as suitable membranes for syngas reforming,^26^ lately also proposed as suitable supports for single atom electrocatalysts in *e.g.*, the hydrogen evolution reaction (HER).^27^ Although these materials have only been computationally studied, synthetic routes are available to produce flakes or patches by atomic manipulation.^28^ In addition, recent experimental advances have demonstrated the successful synthesis of the γ-graphyne suggesting the feasibility of forming *sp–sp* ^2^ hybridized structures with acetylenic linkages,^29^ thereby supporting the potential synthesis of grazynes.

By expanding the grazynes applications universe, their potential use in water desalination, a promising avenue for sustainable freshwater production, remains unexplored. This work addresses this point explicitly, showing the efficiency of grazynes as desalination membranes by conducting a dynamic study of the process, evaluating water permeability and ion rejection performance across different grazyne structures.

## 2 Computational Details

Five grazyne structures with different pore sizes have been considered to evaluate how the pore size affects the passage of water molecules. Previous studies on graphene have indicated that water can consistently permeate membranes with pore diameters smaller than 1 nm.^30^ To this end, five different nanoengineered grazyne structures were designed, each featuring a different pore size. The studied geometries were optimized using the Vienna *ab initio* simulation package (VASP)^31^ through periodic density functional theory (DFT) simulations. The core electron density was modeled using the projector augmented wave (PAW) method,^32^ while the valence electron density was represented using a planewave basis set with a kinetic energy cutoff of 415 eV, ensuring adsorption energy accuracy up to be below the chemical accuracy of 1 kcal·mol^–1^, *ca.* 0.04 eV.^33,34^ To properly incorporate exchange-correlation (*xc*) effects, the Perdew–Burke–Ernzerhof (PBE) *xc* functional was utilized.^35^ Furthermore, Grimme’s D3 method (PBE-D3) was applied to effectively account for dispersive force interactions.^36^ Optimal Monkhorst–Pack **k**-point grids of 10 × 10 × 1 dimensions were used with the electronic and ionic convergence criteria thresholds set at 10^–6^ eV and 10^–5^ eV, respectively. The grazynes selected for the study are shown in Figure 1. Each structure contains a single graphene stripe, with differences arising from the length of the acetylenic bonds and the number of vacancies in this region. Following the nomenclature previously reported,^21,23^ the studied grazynes, from left to right, are [1],[2]{2}-, [1],[1]{2}-, [1],[2]{1}-, [1],[2]{3}-, and [1],[3]{2}-grazyne. Within this notation, the first [1] indicates the presence of a single graphene stripe, the second index, [1], [2] or [3], denotes the length of the acetylenic linkage (*i.e.*, the number of *sp* C pairs), and the last one, {2} or {3}, represents the number of vacancies in the acetylenic region. According to the last index, each of these grazynes exhibits a different pore size, which, after geometric optimization and considering the van der Waals radius of each atom, is presented in Table 1. It should be noted that the grazyne modifications made correspond to the smallest structural changes that can be carried out progressively, that is creating an acetylenic vacancy or enlarging the acetylenic stripes by an extra triple bond. Grazynes with intermediate pore sizes could be obtained (*e.g.*, a pore size between 9 and 15 Å) through functionalization of the pores using larger substituents instead of H. However, these modifications would also alter the local chemistry of the pore, making the comparison no longer purely based on the pore size effects.

**Figure 1:**
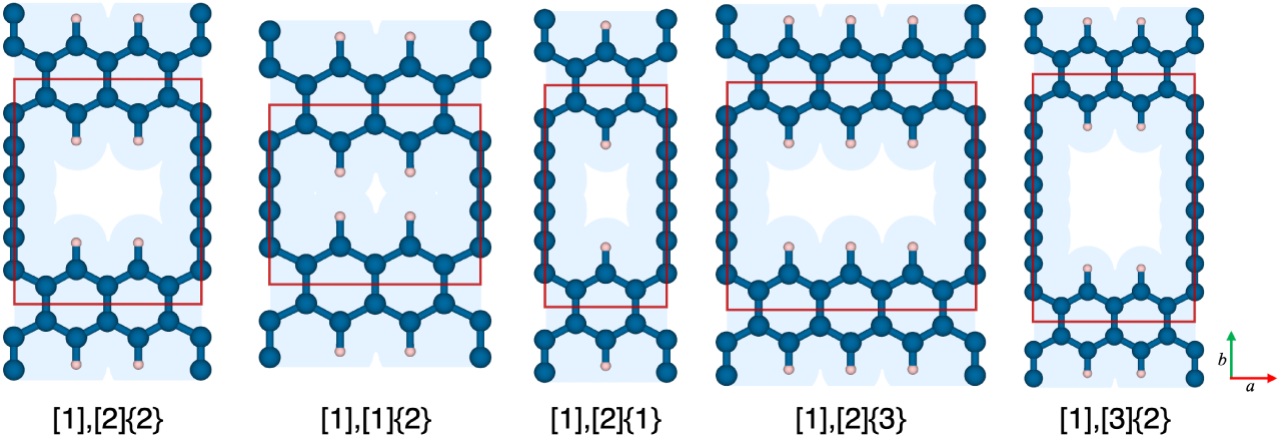
Top view of all grazyne structures considered in this work. Blue spheres represent carbon atoms, while white spheres indicate hydrogen atoms. Red lines outline the primitive unit cell. The light blue background denotes the atomic occupancy when van der Waals radii are taken into account; thus, the white region inside the pore denotes the effective pore size of each grazyne.

**Table 1:** Pore Area Size and Lattice Parameters, *a* and *b*, of Each Considered Grazyne

Grazyne	Pore surface/Å^2^	*a*/Å	*b*/Å
[1],[2]{2}	9.55	7.66	18.57
[1],[1]{2}	1.12	7.64	13.17
[1],[2]{1}	3.72	5.05	18.57
[1],[2]{3}	15.67	10.03	18.46
[1],[3]{2}	20.59	7.55	23.62

Once the optimized geometries were obtained, classical molecular dynamics (MD) simulations of the water desalination process were performed. These simulations were carried out using the large-scale atomic/molecular massively parallel simulator (LAMMPS) package,^37^ and following the same procedure, placing the grazynes at the midpoint of the *z*-axis of the simulation box, thereby creating two sections of equal volume. Since grazynes consist only of C and H atoms, the AIREBO force field^38^ was used to define their interactions. This force field is well-known for accurately reproducing interatomic interactions in C-based materials, as demonstrated on graphene,^39–41^ graphynes,^42,43^ and grazyne membranes.^23–26^ The intermolecular interactions were modeled by Lennard-Jones 12–6 potential plus a Coulomb interaction term,^44^ see eq 1, as

1 $$V_{non-bonded}\left(r_{ij}\right)=\frac{q_{i}q_{j}}{4\pi \varepsilon_{0}r_{ij}}+4\varepsilon_{ij}\left({\left(\sigma_{ij}/r_{ij}\right)}^{12}-{\left(\sigma_{ij}/r_{ij}\right)}^{6}\right)$$

where *ε*_0_ is the vacuum permittivity, *ε*_*ij*_ and σ_*ij*_ are the Lennard-Jones parameters of the *ij* interaction, *r*_*ij*_ the interatomic distance between pairs, and *q*_*i*_ and *q*_*j*_ the partial atomic charges of the *ij* pair species. Note that in the case of interactions between like atoms (*e.g.*, O–O), the subscripts are denoted as *ii* instead of *ij*. The values used to simulate the behavior of H_2_O molecules are defined in Table 2. These were modeled using the well-known TIP4P model with a long-range Coulombic solver,^45^ where the H–O bond distance is set to 0.9572 Å, and the H–O–H angle is 104.52°. The distance between the dummy site and the oxygen atom is 0.1250 Å. A charge of +0.52422 *e* was assigned to each hydrogen atom, balanced by a charge of −1.04844 *e* on the dummy atom.

**Table 2:** Non-Bonded Parameters for H_2_O Using the TIP4P Model^a^

Molecule	Interaction, *ij*	*ε*_*ij*_ /kcal·mol^–1^	σ_*ij*_/Å
H_2_O	O–O	0.16275	3.16435
	O–H	0.0	1.0
	O–D	0.0	1.0
	H–H	0.0	1.0
	H–D	0.0	1.0

a O–O represents the interaction between oxygens, O–H the interaction between oxygen and hydrogen, O–D the oxygen-dummy interaction, H–H the interaction of hydrogens, and H–D the interaction between hydrogen and the dummy atom.

Regarding the dissolved salts in water, only NaCl was considered, being the most abundant salt in seawater, with concentrations of *ca.* 19.35 g/kg for Cl^–^ and 10.78 g/kg for Na^+^.^46^ For this purpose, Na^+^ and Cl^–^ ions were modeled using the parameters listed in Table 3.^47^ The unlike pair parameters were calculated using the arithmetic Lorentz–Berthelot combination rules. During the MD simulations, the H_2_O molecules were treated as rigid entities. Regarding grazynes, their position along the *z*-axis was fixed to prevent undesired movements, while allowing atomic displacements in the *x* and *y* directions. This approach aimed to better capture a more realistic behavior of the membrane. All MD simulations were conducted under the canonical *NVT* ensemble, where the number of particles, *N*, the volume, *V*, and the temperature, *T*, of the system were fixed. Different values of pressure were studied to assess its contribution in the process. To control the temperature of the system and maintain it constant throughout the simulation, the Nosè-Hoover thermostat was employed, as a common, solid option.^48–50^

**Table 3:** Non-Bonded Parameters for Na^+^ and Cl^–^ Where *q*_*i*_ Represents the Partial Charge of the Ion, and *ε*_*i*_ and σ_*i*_ the Lennard-Jones Parameters

Ion	*q*_*i*_/*e*	*ε*_*i*_ /kcal·mol^–1^	σ_*i*_/Å
Na^+^	+1	0.1684	2.2589
Cl^–^	–1	0.0117	5.1645

With these parameters defined, the MD simulations were carried out following the same procedure for each grazyne structure to evaluate water desalination through them. Two separate sections were created, divided by the grazyne membrane: One containing water molecules and ions, and the other containing only pure water molecules. In the saline water section, a piston was placed at the top to apply pressure and drive the transport of molecules toward the pure water section. All simulations began with a 300 ps thermalization process to ensure that the mixture stabilized at 300 K. During this period, molecules and ions were prevented from interacting with the grazynes by two walls positioned just above and below them. Once thermalization was complete, the piston was gradually moved until the desired pressure was reached. This process was carried out in multiple phases to prevent an excessive temperature increase in the mixture. This procedure was repeated until the desired pressure was achieved. Once the pressure was reached, the two mentioned walls were removed, allowing the interaction between grazyne and water while enabling the passage of molecules and/or ions through the membrane. The diffusion process was then maintained for 100 ps, although in some cases the simulation time was extended 300 ps to ensure convergence. Four different pressure values were studied for each type of grazyne, with three independent replicates performed for each pressure, resulting in a total of 12 simulations per structure. This approach ensures more representative results, minimizing the influence of the stochastic nature of the process. The pressures analyzed were set close to 150, 170, 190, and 210 MPa. Although these values are significantly higher than those used in conventional reverse osmosis systems, they are employed here to accelerate permeation events and obtain statistically meaningful transport data within accessible simulation time scales. Therefore, the present simulations should be interpreted primarily as a mechanistic exploration of water and ion transport through grazyne membranes rather than as a direct representation of industrial operating conditions. Previous MD and DFT studies on grazyne membranes have shown that these materials preserve their structure under dynamical conditions.^23–26^ Although the membranes exhibited flexible wave-like fluctuations during simulations, no loss of the intrinsic grazyne morphology was observed. Figure 2 presents a schematic representation of the simulation box. For each grazyne, a fixed number of molecules was used to maintain consistency across all conditions, and a NaCl concentration of *ca.* 35 g/L was modeled, matching the salinity of seawater.

**Figure 2:**
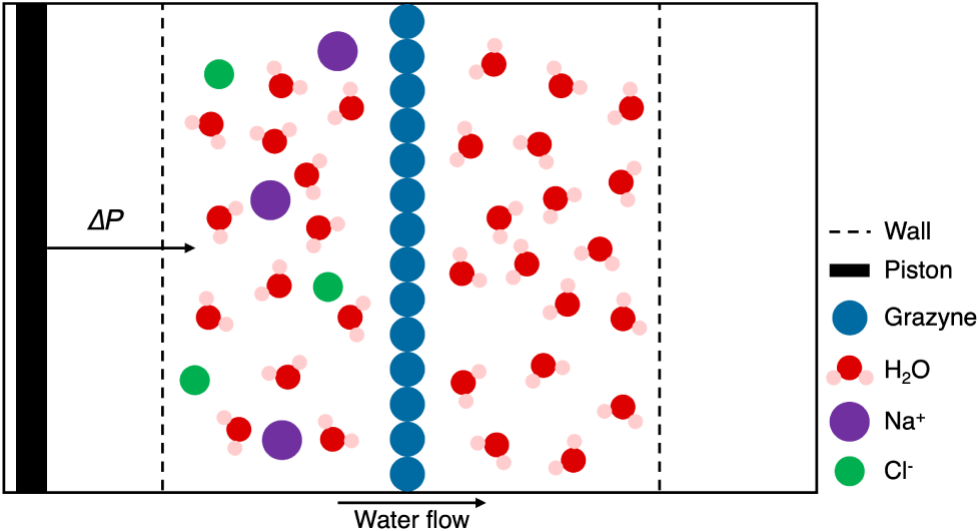
Schematic side view of the simulation box. A piston applies a pressure difference, Δ*p*, on the feed side, containing H_2_O molecules, red sphere for O and white for H, together with Na^+^, purple, and Cl^–^, green, ions. The grazyne membrane, colored as blue spheres, separates both sides. Dashed lines indicate the confining walls of the simulation.

The permeability of H_2_O through the membranes was obtained directly from the results obtained in the MD simulations. For each applied pressure, Δ*p*, the total number of H_2_O molecules that crossed the membrane, Δ*N*, was calculated during the simulation time, *t*. With this data, the total volume of water filtered, *V*_filtered_, can be calculated as

2 $$V_{\mathrm{filtered}}=\Delta N\cdot V_{\mathrm{mol}}$$

where *V*_mol_ is the volume associated with a single H_2_O molecule and is obtained using the following expression:

3 $$V_{\mathrm{mol}}=\frac{M_{H_{2}O}}{\rho \cdot N_{A}}$$

where $M_{H_{2}O}$ is the mass of the H_2_O molecule, *N*_*A*_ is Avogadro’s constant, and ρ is the density of liquid H_2_O where a value of 1 g/cm^3^ was assumed. From *V*_filtered_, it is possible to calculate the volumetric flow rate as

4 $$Q=\frac{V_{\mathrm{filtered}}}{t}$$

where *t* is the simulation time of the diffusion process. In order to carry out a representative comparison between the different grazynes, Q was normalized by the simulation cell area, *A*_cell_, as follows:

5 $$J=\frac{Q}{A_{\mathrm{cell}}}$$

Then, the permeability of H_2_O, $P_{H_{2}O}$ , is calculated as

6 $$P_{H_{2}O}=\frac{J}{\Delta p}$$

For further details on how the permeability was calculated, we refer the reader to Section S1 of the Supporting Information (SI). Finally, the salt rejection, SR, was computed as follows:

$$SR=1-\frac{N_{1/2}}{N_{0}}$$ 7

where *N*_1/2_ is the number of ions permeated after half of the water molecules crossed the membrane and N_0_ the total number of ions.

## 3 Results and Discussion

Simulations were carried out for the five grazynes shown in Figure 1, considering four different pressures and three replicates for each pressure. The results are summarized in Table 4. Grazynes with the smallest pores, *i.e*. [1],[1]{2} and [1],[2]{1}, are not included in the table since they featured neither H_2_O molecules nor ion permeation during the MD simulations. To be exact, [1],[1]{2} did not allow the passage of any species at any of the three pressures studied, and [1],[2]{1} only managed to filter four H_2_O molecules at 210 MPa. Based on these results, neither of these two grazynes is suitable for water desalination, and so their structures were therefore modified to increase the pore size. This increase led to a significant improvement in the membrane permeability. In this sense, the three new grazynes showed promising performance in terms of H_2_O transport. At the lowest pressure studied, *i.e.*, 150 MPa, neither of the three structures — [1],[2]{2}, [1],[2]{3} and [1],[3]{2} — exhibited significant H_2_O permeability, a trend that changes when the pressure is increased by 20 MPa. At 170 MPa, structure [1],[3]{2} exhibited a higher flow rate compared to the other structures. An average of 91 water molecules permeated, representing 6.6% of the H_2_O molecules in the saltwater region. As for the other two grazynes, [1],[2]{2} and [1],[2]{3}, both showed slightly lower water flow at this pressure, with average values of 22 and 36 permeated water molecules, respectively. Similar to [1],[3]{2}, neither of these two allowed any salt ions to pass through. When the pressure was increased to 190 MPa, the trend was maintained, with the [1],[3]{2} structure being the grazyne with the highest water flow by far, reaching an average of 356 filtered H_2_O molecules and representing 25.9% of the saltwater. On the other side, both [1],[2]{2} and [1],[2]{3} also increased the flow rate achieving 10.9% and 11.2% of the saltwater filtered, respectively. The increased flow was accompanied by a decrease in the salt rejection in all grazynes. In particular, [1],[3]{2} showed an average permeation of *ca.* 4 ions for the two highest pressures, *i.e.*, 190 and 210 MPa. For the [1],[2]{2}, only the passage of Na^+^ was observed for the two pressures mentioned. Finally, at a pressure of 210 MPa, the same trend observed at 190 MPa was seen. Both [1],[2]{3} and [1],[3]{2} showed the highest water flow rate, with an average of 264 and 492 H_2_O molecules filtered, respectively.

**Table 4:** Average of the Number of H_2_O Molecules and Na^+^ and Cl^–^ Ions Filtered at the End of the Simulations at Four Different Pressures^a^

Grazyne	[1],[2]{2}				[1],[2]{3}				[1],[3]{2}			
*p*/MPa	150	170	190	210	150	170	190	210	150	170	190	210
H_2_O	1	22	129	249	1	36	135	264	6	91	356	492
Na^+^	0	0	0.3	0.3	0	0	0	0.7	0	0	3.2	3.6
Cl^–^	0	0	0	0	0	0	0	0.3	0	0	4	4.3
**H**_**2**_ **O%**	**0.1**	**1.8**	**10.9**	**21.0**	**0.1**	**3.0**	**11.2**	**22.1**	**0.4**	**6.6**	**25.9**	**35.7**

a The value represents the average of the three replicates carried out for each pressure.

However, the results suggest that [1],[2]{3} offers better salt rejection, as an average of 0.7 Na^+^ and 0.3 Cl^–^ ions were able to pass through it. In contrast, [1],[3]{2} allowed up to 3.6 and 4.3 ions to pass, indicating that its pores might facilitate ion transport to a greater extent. As for the last grazyne, the [1],[2]{2}, it obtained an average of 249 H_2_O molecules filtered with an average of 0.3 Na^+^ ion permeated. However, even with a certain ion flow, the resulting water can still be used. The WHO considers salinity primarily an organoleptic parameter and not a direct health risk, except in cases of extreme conditions. In this regard, the WHO recommends that water with total dissolved solids (TDS) values below 600 mg/L is considered good quality and has excellent taste and considers water with TDS values in the 900–1200 mg/L range to have poor taste.^51^ For [1],[2]{3}-grazyne at 210 MPa, a permeate salinity of 807 mg/L was obtained, a value that falls within the range of water considered to have poor taste. Despite the limitations on direct consumption, it is important to evaluate the potential use of this permeate for basic water supply, or in agriculture. The salinity obtained from TDS corresponds to *ca.* an electrical conductivity (EC) of 1.21 mS·cm^–1^. According to the guidelines of FAO,^52^ water with an EC of 1.21 mS·cm^–1^ (*i.e.*, 1210 μS·cm^–1^) is classified as medium risk and is generally considered suitable for irrigating a large number of crops, suggesting a viable application of the process. Although the single-pass TDS value exceeds the WHO organoleptic standards, the process can be improved in a two-stage or multipass desalination system, where a second filtration process would likely bring the salinity down to better taste levels. A more visual inspection of the results can be found in Figure 3, where the number of H_2_O molecules filtered at the end of the simulation is represented, with their respective error bars, as a function of pressure. From it, [1],[3]{2}-grazyne clearly obtained a higher water flow than the rest of grazynes.

**Figure 3:**
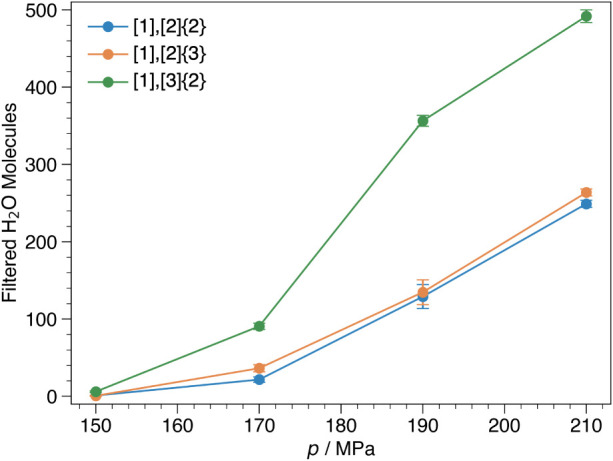
Number of H_2_O molecules permeated at the end of the simulations for each studied pressure, including error bars. Blue spheres correspond to [1],[2]{2}-grazyne, orange to [1],[2]{3}-grazyne, and green spheres to [1],[3]{2}-grazyne.

Figure 4 shows the temporal evolution of the number of H_2_O molecules crossing the membranes for the three highest studied pressures, as well as with three independent replicates in order to quantify both the variability of the structure itself and the reproducibility of the process. Permeation was simulated for up to 100 ps, which was enough to achieve convergence in transport properties; in selected cases, an additional time of 300 ps was simulated to confirm this (see Section S2 in the SI).

**Figure 4:**
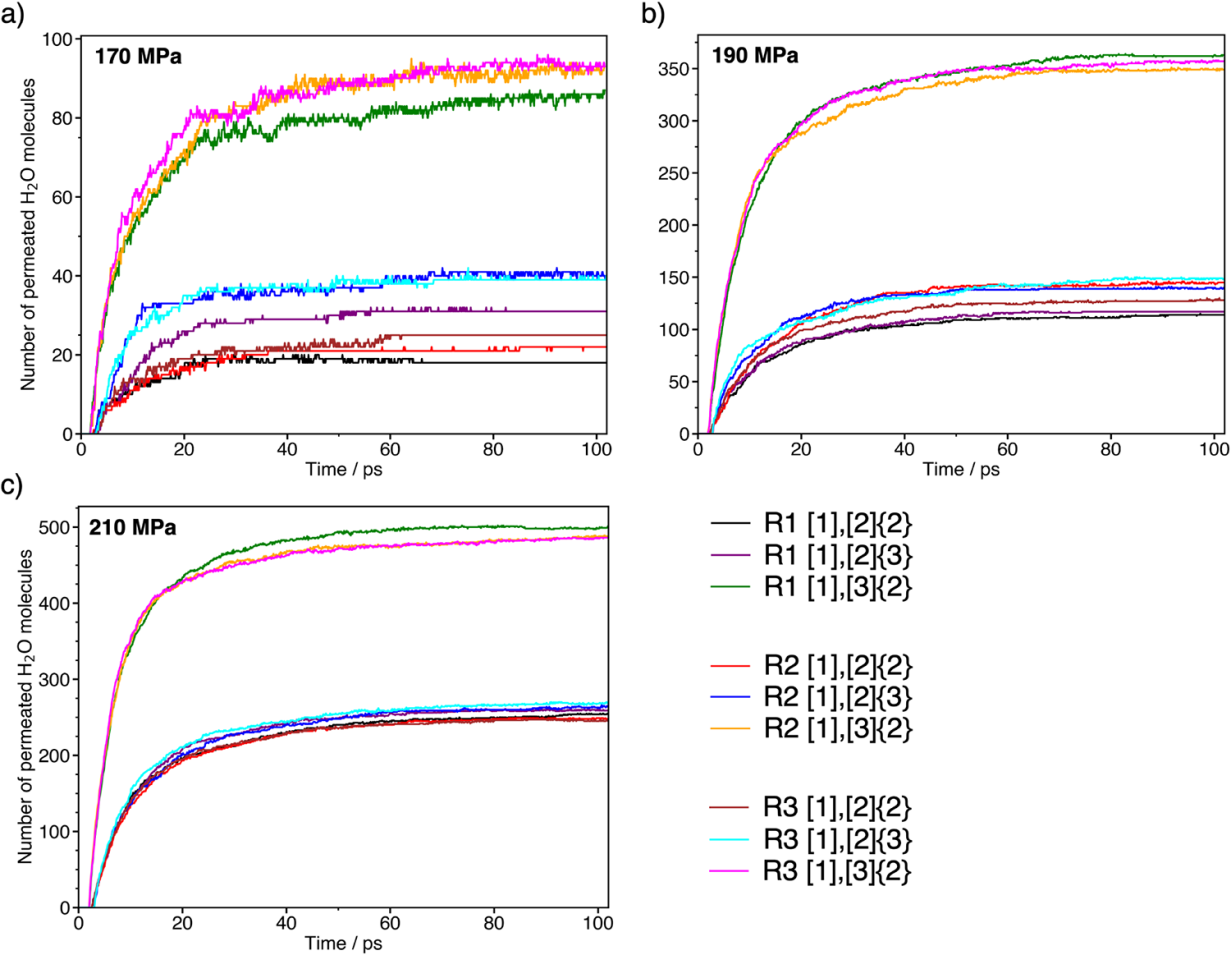
Number of H_2_O molecules permeated through a) [1],[2]{2}-, b) [1],[2]{3}-, and c) [1],[3]{2}-grazyne under 170, 190, and 210 MPa. Three simulation replicates for each structure and pressure are shown.

The results for the pressure of 150 MPa are not shown due to the low permeability observed, see Table 4. From Figure 4, it is clear that water transport is strongly dependent on the pressure applied in the process. At 170 MPa, permeation remained low throughout the studied time range, reaching final values of a few tens of molecules, while at 190 MPa the number of H_2_O molecules permeated increased substantially, exceeding 300 molecules for the [1],[3]{2}-grazyne. This increase was even more pronounced at 210 MPa, where all membranes systematically obtained a much higher water flow, exceeding 200 molecules in [1],[2]{2} and [1],[2]{3} and 400 in the [1],[3]{2} structure. The differences between membranes are also consistent across the entire conditions studied. As already mentioned, [1],[3]{2}-grazyne allows significantly greater permeation, which is reflected in steeper initial slopes. The fact that these differences persist when the pressure is increased indicates that both the geometry and chemistry of the pore significantly influence water flow. Regarding the reproducibility, replicates show little variability among them. At all pressures, a fast initial kinetic zone is observed, followed by a stationary phase in which the number of permeated molecules increases slowly. This behavior is characteristic of confined diffusion processes and reflects the dynamic equilibrium of the system under study. It is important to emphasize that this process is stochastic, so the results will strongly depend on the initial configuration of the system — the arrangement of the membrane and water molecules — and therefore, obtaining replicates with similar results provides a solid basis for analyzing the results.

With values in Table 2 and using eq 6, one can compute the $P_{H_{2}O}$ of each grazyne as a function of the applied pressure and the results are shown in Figure 5. To avoid overestimating the flow due to the rapid initial permeation caused by the pressure gradient, the initial section of each trajectory was excluded until a linear trend was observed. From it, all grazynes share the same trend, permeability increases with pressure, confirming that water flow is strongly driven by the pressure gradient. This increase is almost linear for all grazynes. The three membranes exhibited different permeability values within the studied pressure range, highlighting the importance of the morphology and chemistry of the pore. In particular, the [1],[2]{2}-grazyne shows the greatest increase with pressure, reaching the highest permeability at 210 MPa, while the [1],[2]{3}-grazyne is consistently the least permeable. The apparent deviation from the linear trend for the [1],[3]{2}-grazyne at 210 MPa and for the [1],[2]{2}-grazyne at 170 MPa can be attributed to the stochastic nature of the permeation process. Given the limited number of replicates, the final average is highly sensitive to statistical fluctuations in individual trajectories. In addition to water permeability, Figure 6 also shows the corresponding salt rejection values for the three membranes. Increasing pore size improves water transport, especially at high pressures, but simultaneously allows larger passage of ions, thus reducing salt rejection. In this regard, the [1],[2]{2}-grazyne preserves almost complete ion rejection throughout the entire pressure range while exhibiting significant water permeabilities. On the other hand, the [1],[3]{2}-grazyne achieves the highest water permeability, although at the expense of a reduction in selectivity, especially at 190 and 210 MPa, while the [1],[2]{3}-grazyne displays an intermediate behavior, suggesting a more balanced compromise between permeability and selectivity. The difference between results confirms that the specific morphology of each membrane controls the ease with which molecules pass through the pores. Combining these results with those in Table 4, [1],[2]{2}-grazyne would be the most suitable to carry out the separation since it is the one that obtained a greater permeation of water molecules with a greater salt rejection, having only filtered 0.3 Na^+^ ions, which would translate into an almost perfect desalination at both 170 and 210 MPa. The values reported here should be interpreted as estimates of the intrinsic transport properties of the membranes rather than exact predictions under low-pressure operating conditions, where deviations from linearity could in principle arise.

**Figure 5:**
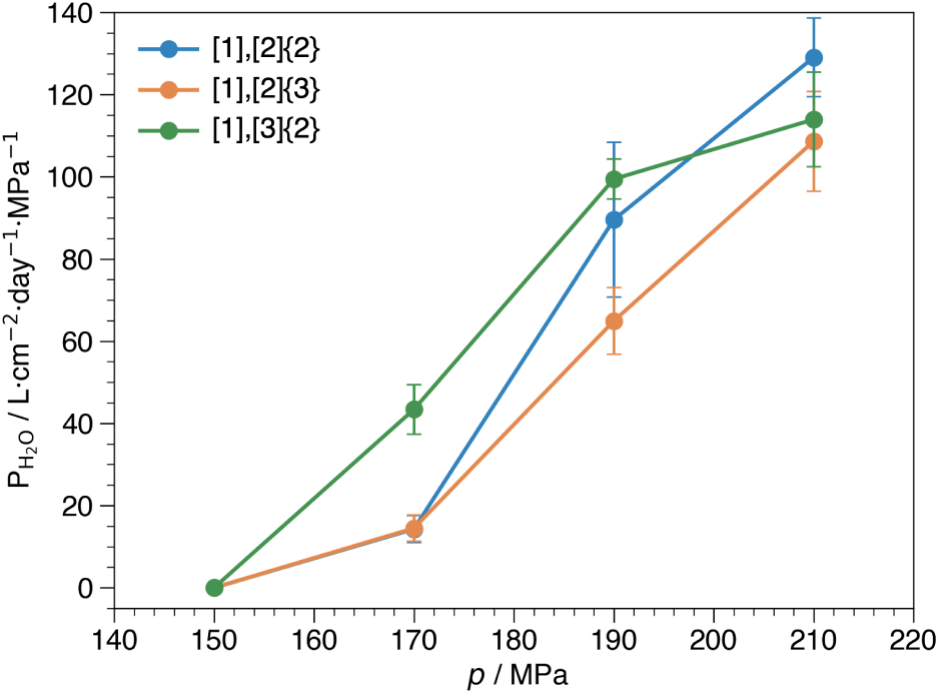
Water permeability for the three studied grazynes as a function of the applied pressure – 150, 170, 190, and 210 MPa, including error bars. Blue spheres correspond to [1],[2]{2}-grazyne, orange to [1],[2]{3}-grazyne, and green spheres to [1],[3]{2}-grazyne. Each data set represents the average permeability from three independent simulations.

**Figure 6:**
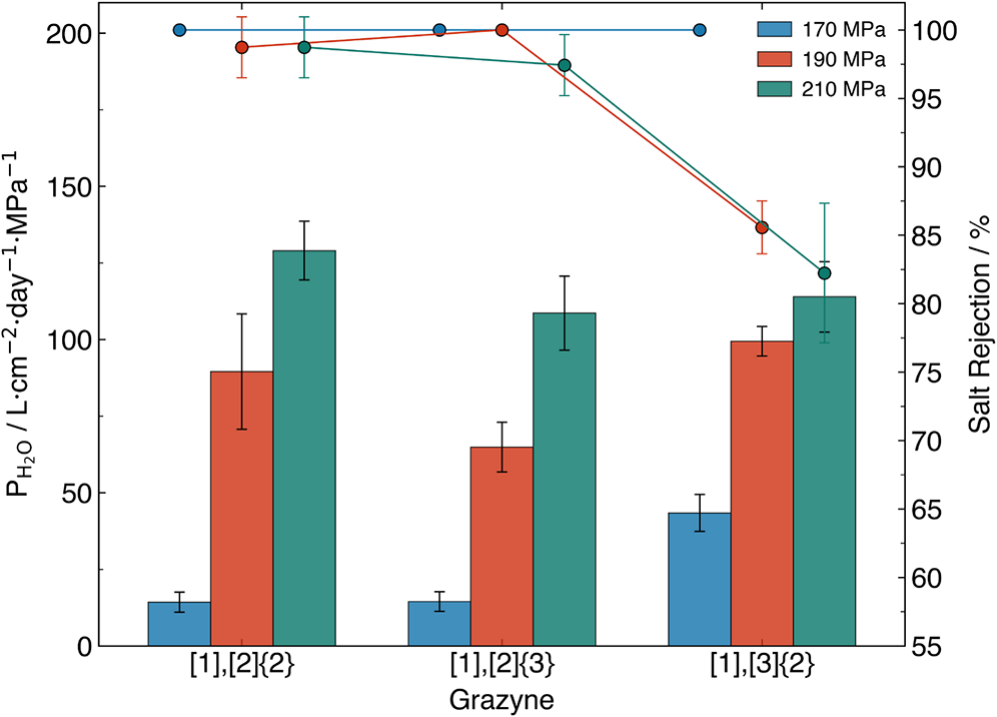
Water permeability (bars, left axis) and salt rejection (lines, right axis) for the different grazyne membranes at 170, 190, and 210 MPa in, blue, orange, and green, respectively.

As previously reported, water transport through grazynes is strongly governed by pore geometry and increases systematically with the applied pressure. This is reflected once again in Table 5, which summarizes the water and ion fluxes through the three studied grazynes. To calculate the flux, the net passage of molecules through the membranes was considered, *i.e.*, the molecules crossing the membrane in both directions were counted, and the net value was calculated from these two values. In all cases, the water flux is significantly higher than those for Na^+^ and Cl^–^, a finding expected after analyzing the previous results. This fact confirms the high selectivity of these membranes toward water. Specifically, for the [1],[2]{2}-grazyne, almost no permeation was detected at 150 MPa, while at 170 MPa a very low flux appears, increasing rapidly to 0.86 ps^–1^ at 210 MPa. Ion transport is practically nonexistent, with only a very low Na^+^ flux appearing above 190 MPa and zero Cl^–^ flux across the entire pressure range. The [1],[2]{3}-grazyne exhibits similar behavior, with a permeation close to zero at 150 MPa. Water flux at 210 MPa reaches values comparable to those of the previous grazyne, with a value of 0.98 ps^–1^. The flux of 0.01 ps^–1^ for both Na^+^ and Cl^–^ suggests a wider permeability range but is still highly selective toward water. Finally, [1],[3]{2}-grazyne showed the highest permeability, showing water flux even at 150 MPa. This higher permeability is accompanied by the appearance of traces of both Na^+^ and Cl^–^ at high pressures, indicating that a larger pore size clearly favors the passage of ions, although their flux remains very low compared to water. It is worth noting that the grazyne membranes considered in this work are electrically neutral, as they are composed exclusively of carbon and hydrogen atoms without any functionalization. Therefore, ion rejection is not driven by long-range electrostatic exclusion mechanisms. Instead, the observed selectivity arises primarily from geometric restrictions and dehydration effects. Hydrated ions such as Na^+^ and Cl^–^ would need to partially reduce their hydration shells to permeate through the pores, which is energetically unfavorable and reduces their transport.

**Table 5:** Flux Rate of Water, Na^+^ and Cl^–^ across Grazynes^a^

Grazyne	[1],[2]{2}				[1],[2]{3}				[1],[3]{2}			
*p*/MPa	150	170	190	210	150	170	190	210	150	170	190	210
H_2_O	0.01	0.08	0.55	0.86	0.01	0.15	0.54	0.98	0.06	0.31	0.78	0.99
Na^+^	—	—	0.01	0.01	—	—	—	0.01	—	—	0.04	0.04
Cl^–^	—	—	—	—	—	—	—	0.01	—	—	0.05	0.05

a Units in molecule/ps

By analyzing the trajectories of water molecules, it has been possible to identify the pore regions where molecules pass most frequently — the most accessible pore regions. Figure 7 shows the relative water oxygen density right at the pore, based on the results at 210 MPa of pressure. In the case of [1][2]{2}-grazyne, the density is concentrated at the center of the pore, making the effective pore region very small. This indicates that molecules tend to follow specific localized trajectories crossing the membrane through a single channel. In the [1],[2]{3} membrane, where the pore is wider, the density is more spread out along the *x* direction of the pore. Instead of a single point of high density, a continuous band is observed, indicating multiple and less restricted trajectories. Still, the center of the pore remains the region of highest density, as it is the point furthest from the lateral acetylenic strips, thus avoiding steric repulsions. Finally, [1],[3]{2}-grazyne shows a highly concentrated density in two different zones of the pore. This behavior indicates that water transport is primarily organized through these two zones. Comparing the [1],[2]{2}- and [1],[3]{2}-grazynes, which differ in the length of their acetylenic strips but contain the same number of hydrogens, it can be seen how enlarging the pore allows molecules to possess not only two well-defined channels but also longer channels along the *x*-axis, thus facilitating their passage. The densities obtained agree perfectly with the flux rates values obtained, demonstrating that with a larger pore size, the effective pore region increases, thereby creating different channels for molecules to cross the membranes. Furthermore, the densities are consistent with the effective pore size shown in Figure 1. This confirms that using the van der Waals radii of the atoms accurately predicts which area of the pore is accessible to the molecules.

**Figure 7:**
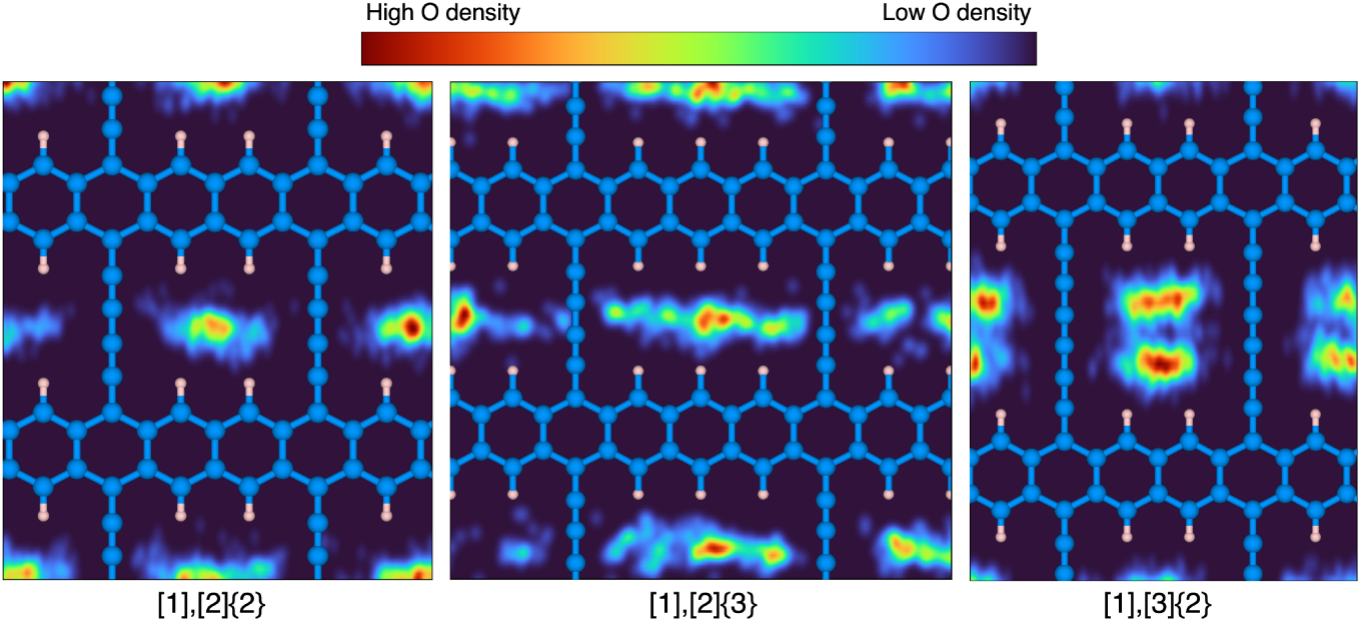
Relative water oxygen density map inside different grazyne pores, with atomic models color-code as in Figure 1. Dark blue and red indicate regions with the lowest and highest probabilities of finding an O atom, respectively.

To better understand the transport mechanism of water molecules, the number of water molecules found within the pores was analyzed throughout the simulations. The probability of the resulting occupancy is shown in Figure 8, revealing a clear dependence on the pore size of each membrane. For the [1],[2]{2} and [1],[2]{3}-grazynes, those with the smallest pores, the distribution is centered on only one molecule in the pore with a low contribution from double-molecule passages in the [1],[2]{3}. This fact indicates strong confined transport where molecules cross the membrane sequentially, in a single-file-like manner. On the other hand, for the grazyne with the largest pore, [1],[3]{2}, the distributions broaden significantly, with some probability of finding two or more molecules simultaneously within the same pore, reflecting a multimolecule transport. These findings are consistent with the oxygen density maps shown in Figure 7 where, in the case of narrow pores, the density is highly localized in a single channel, indicating a well-defined transport path. As the pore size increases, the density becomes more delocalized and generates two different channels, consistent with the simultaneous presence of multiple molecules and a less constrained transport pathway.

**Figure 8:**
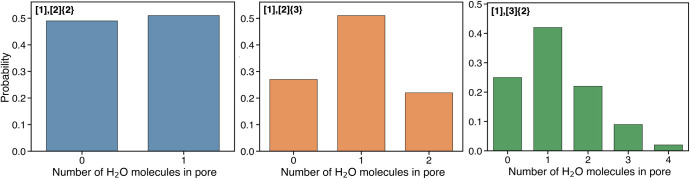
Probability distribution of the number of water molecules inside the same pore for the three systems studied: [1],[2]{2} (left), [1],[2]{3} (center), and [1],[3]{2} (right).

Together, the oxygen density maps, the pore occupancy distributions, and the potential mean force (PMF) profiles provide a consistent and complementary mechanistic picture of water transport across grazyne membranes. The density maps reveal where molecules preferentially cross; the occupancy distributions characterize whether transport is sequential or collective; and the PMF profiles (Figure 9) quantify the energetic cost associated with each regime. Specifically, the PMF of water molecules shows that the [1],[2]{2}-grazyne displays a well-defined central peak at the membrane plane (*z* = 0), reaching *ca.* 8.5 kJ·mol^–1^ at 170 MPa, accompanied by two minima of *ca.* −1.5 kJ·mol^–1^ on either side. These minima correspond to preferred adsorption positions of water molecules immediately before and after crossing the membrane, reflecting a clear interaction with the membrane. The height and narrowness of the central barrier are consistent with the single-file transport regime identified from the oxygen density distributions, see Figures 7 and 8. Moreover, the barrier height decreases slightly as pressure increases — from *ca.* 8.5 kJ·mol^–1^ at 170 MPa to *ca.* 7.5 kJ·mol^–1^ at 210 MPa. The [1],[2]{3}-grazyne exhibits a similar PMF profile, with a central maximum of *ca.* 8 kJ·mol^–1^ at 170 MPa decreasing to *ca.* 7.5 kJ·mol^–1^ at 210 MPa. The pressure dependence of the barrier follows the same trend as for [1],[2]{2}. The most different profile is observed for the [1],[3]{2}-grazyne. Here, the central peak reaches only *ca.* 5 kJ·mol^–1^ and is considerably broader than those of the two structures with smaller pores. The flattening of the free energy across the pore region is consistent with the multimolecule transport behavior identified for this grazyne: the larger pore allows simultaneous passage of two or more water molecules, which lowers the energetic cost of crossing for molecules. The reduced barrier height explains the higher water flux observed for [1],[3]{2} across all pressures studied, as well as the appearance of ion traces at high pressure. Clearly, as the pore size increases from [1],[2]{2} to [1],[3]{2}, the central energy barrier progressively decreases from 8.5 to 5 kJ·mol^–1^. The optimal pore size identified for grazynes falls within the range previously reported for efficient graphyne desalination membranes.^56^ This agreement suggests that similar physical constraints govern water transport across different nanoporous C-based structures. At the same time, in grazynes, pore size modification directly controls the water flux, from strongly single-channel permeation in the smaller pores to more permissive multimolecule transport in the larger ones.

**Figure 9:**
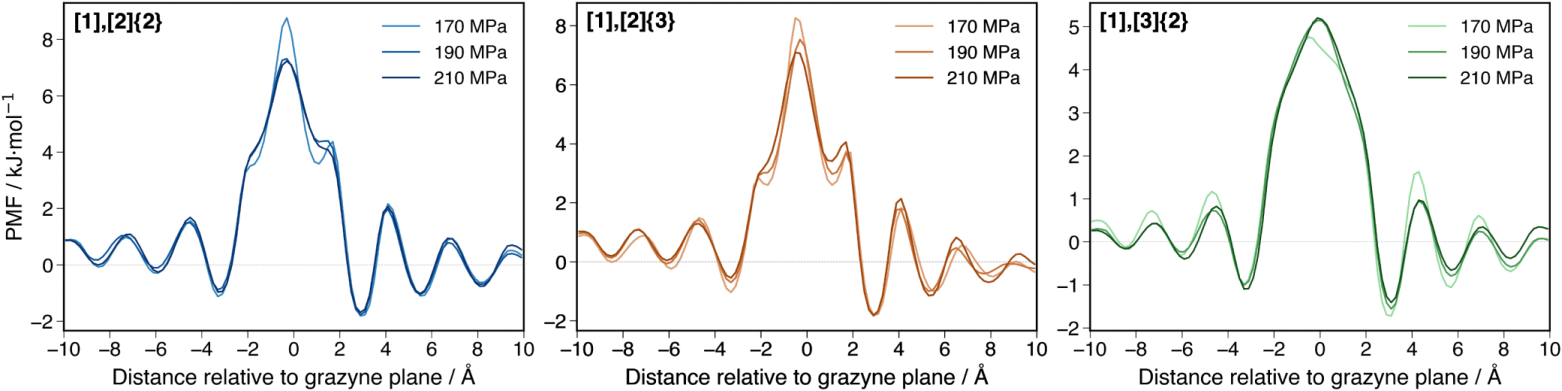
Potential mean force (PMF) along the membrane coordinate *z* for water molecules crossing the [1],[2]{2} (left), [1],[2]{3} (center), and [1],[3]{2}-grazyne (right) membranes. The membrane plane is located at *z* = 0. Curves correspond to applied pressures of 170 MPa (light color), 190 MPa (medium color), and 210 MPa (dark color).

To better understand the transport of each species across grazynes, the passage of Na^+^ and Cl^–^ ions across the membranes has been analyzed, both quantitatively and qualitatively. In this sense, additional DFT calculations were performed for the [1],[3]{2}-grazyne at 210 MPa. Representative ion–water configurations were extracted from the MD trajectories both in the bulk solution and at the center of the pore. The resulting geometries are shown in Figure 10. In both cases, the ions evolve from being coordinated by nine water molecules in the bulk to only five water molecules in the pore region, indicating a reorganization of the hydration shell upon permeation. The corresponding DFT energy differences provide an approximate estimate of the energy associated with partial ion dehydration and shell reorganization. Cl^–^ exhibits a dehydration energy of *ca.* 26.05 kJ·mol^–1^, whereas Na^+^ shows a lower value of *ca.* 7.72 kJ·mol^–1^. These values are consistent with previous studies on ion dehydration in nanoporous systems.^53,54^ Interestingly, although the present dehydration energies suggest that Na^+^ should permeate more easily due to its lower energy, the MD simulations reveal a larger number of permeation events for Cl^–^ in this grazyne at this pressure. This indicates that ion dehydration alone does not fully determine ion selectivity. Instead, the overall permeation behavior also depends on additional factors such as adsorption minima within the pore, residence times, and dynamical transport effects. It can also be attributed to the fact that only one case has been studied, making it statistically unrepresentative.

**Figure 10:**
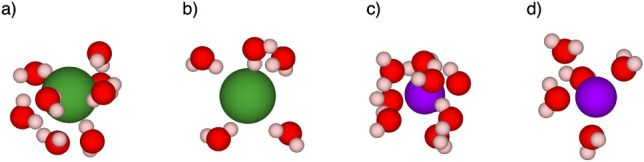
Representative geometries of Cl^‑^ (green) and Na^+^ (purple) with their first solvation shell in (a, c) the bulk region and (b, d) the pore region of the [1],[3]{2}-grazyne at 210 MPa. Oxygen and hydrogen atoms are represented in red and white, respectively.

Moreover, the pathways at 210 MPa were studied, as these are the most permeable simulations to these species, focusing on the pore region. In them, both cations and anions cross the membrane through the center of the pores, as shown in Figures 11 and 12. Furthermore, the approach to the pore occurs both individually and as an ion pair. In this regard, two different behaviors were observed when crossing the membrane as an ion pair: one where the pair crosses the pore together, and another where the approach occurs together but upon passing through the pore, the ions separate and pass individually. An example of the first case is shown in Figure 13. These observations suggest that ion transport is not restricted to a single mechanism, but rather combines individual and cooperative pathways, depending on the local configuration and immediate environment. In this regard, Figures S2–S7 of Section S3 of the SI provide the Radial Distribution Functions (RDF) of the different pairs that are present in the simulations for each permeable grazyne. Cl^–^ clearly shows a stronger interaction with the H atoms of water, leading to two different peaks in the RDFs, the first of which appears at shorter distance than the corresponding Cl^–^-O distance. In contrast, Na^+^ preferentially interacts with O atoms, as expected, since O carries the partial negative charge in the water molecule. The RDFs confirm that solvation in the bulk is consistent for all grazynes at all the applied pressures (*cf*. Figures S2, S4, and S6 of the SI). These bulk region profiles serve as a reference for evaluating the structural changes that the systems undergo when ions approach the pore region. The ion– $O_{H_{2}O}$ RDF of both Na^+^ and Cl^–^ maintains a well-defined first and second solvation shell in the bulk region, with the first shell of Na^+^ centered at *ca*. 2.3 Å and that of Cl^–^ at *ca*. 3.1 Å, and their respective second shells at *ca*. 4.5 and *ca*. 6.0 Å. In bulk, water molecules in the first solvation shell adopt different orientations, giving rise to two distinct ion– $H_{H_{2}O}$ peaks separated by *ca*. 1.5 Å, consistent with the intramolecular H–H distance in water. The first peak corresponds to the hydrogen atom pointing toward the ion, while the second reflects the more distant hydrogen of the same water molecule. This behavior is more evident for the ${\mathrm{Cl}}^{-}-H_{H_{2}O}$ RDF than for ${\mathrm{Na}}^{+}-H_{H_{2}O}$ . In the case of Na^+^, the ion interacts preferentially with the oxygen atom of the water molecules rather than directly with the hydrogen atoms. As a result, the ${\mathrm{Na}}^{+}-H_{H_{2}O}$ RDF contains contributions from a broader range of water geometries beyond the simple near-H/far-H orientation, leading to broader peaks. The RDFs of the pore region (see Figures S3, S5, and S7 of the SI) show structural differences between grazynes that help explain the different trends in ionic rejection observed. In the pore, the second solvation shell of both ${\mathrm{Na}}^{+}-O_{H_{2}O}$ and ${\mathrm{Cl}}^{-}-O_{H_{2}O}$ is substantially reduced, indicating partial dehydration. Consistently, the second peak of both ${\mathrm{Na}}^{+}-H_{H_{2}O}$ and ${\mathrm{Cl}}^{-}-H_{H_{2}O}$ is also reduced in the pore, indicating a reorganization of first-shell water molecules toward a more symmetric configuration. This reduction is more pronounced for Na^+^ than for Cl^–^, for which two relatively well-defined peaks are still observed in the pore region. This in-pore reorganization effect is most evident in the [1],[2]{3}- and [1],[3]{2}-grazynes, which are in fact the structures where the most ion diffusion through the membrane is observed. For the latter grazyne, the ${\mathrm{Cl}}^{-}-O_{H_{2}O}$ pore RDF presents a more diffuse profile compared to the other grazynes, suggesting that when the pore is large enough, Cl^–^ can partially lose its structured hydration shell, and the energy barrier becomes sufficiently reduced to allow its passage. Finally, the Na^+^–Cl^–^ pore RDF for this grazyne also shows a clear first peak and a noisy profile, resulting in a clear ion pair peak, in agreement with the paired and individual crossing mechanisms, as shown in Figures 11, 12, and 13. To further quantify the ion partial dehydration suggested by the RDF profiles, the coordination number, CN, of both ions and the residence time, τ, of the first hydration shell were computed in both the bulk solution and the pore region. The results are reported in Table S2 of the SI. In the bulk solution, Na^+^ coordinates *ca*. 5.8–6.1 water molecules through ${\mathrm{Na}}^{+}-O_{H_{2}O}$ interactions, while Cl^–^ coordinates *ca*. 5.9–6.6 water molecules through ${\mathrm{Cl}}^{-}-H_{H_{2}O}$ interactions. In the pore region, both ions display lower CN values than in the bulk, confirming that ion approach and permeation through the grazyne pores require partial dehydration and some reorganization of the hydration shell. This effect is particularly relevant for Cl^–^ in the [1],[2]{2}- and [1],[2]{3}-grazynes, where the more structured Cl^–^ hydration shell, evidenced by the double-peaked RDF profiles, leads to a higher dehydration and explains the preferential rejection of Cl^–^ over Na^+^. For [1],[3]{2}, the larger pore allows both ions to retain part of their hydration shell inside the pore region, lowering the need for reorganization and allowing Na^+^ and Cl^–^ permeation at high pressure. The residence time analysis indicates that water molecules exchange more rapidly in the pore region than in the bulk, especially for [1],[3]{2}, where ion permeation events are statistically better sampled. This shorter residence time reflects a more labile hydration environment and supports the conclusion that ion passage requires partial dehydration. For the smaller [1],[2]{2}- and [1],[2]{3}-grazynes, the pore CN and τ values for Cl^–^ have larger statistical uncertainty — Cl^–^ almost never crosses these membranes — and should therefore be interpreted as descriptors of the ion pore approach rather than of actual permeation.

**Figure 11:**
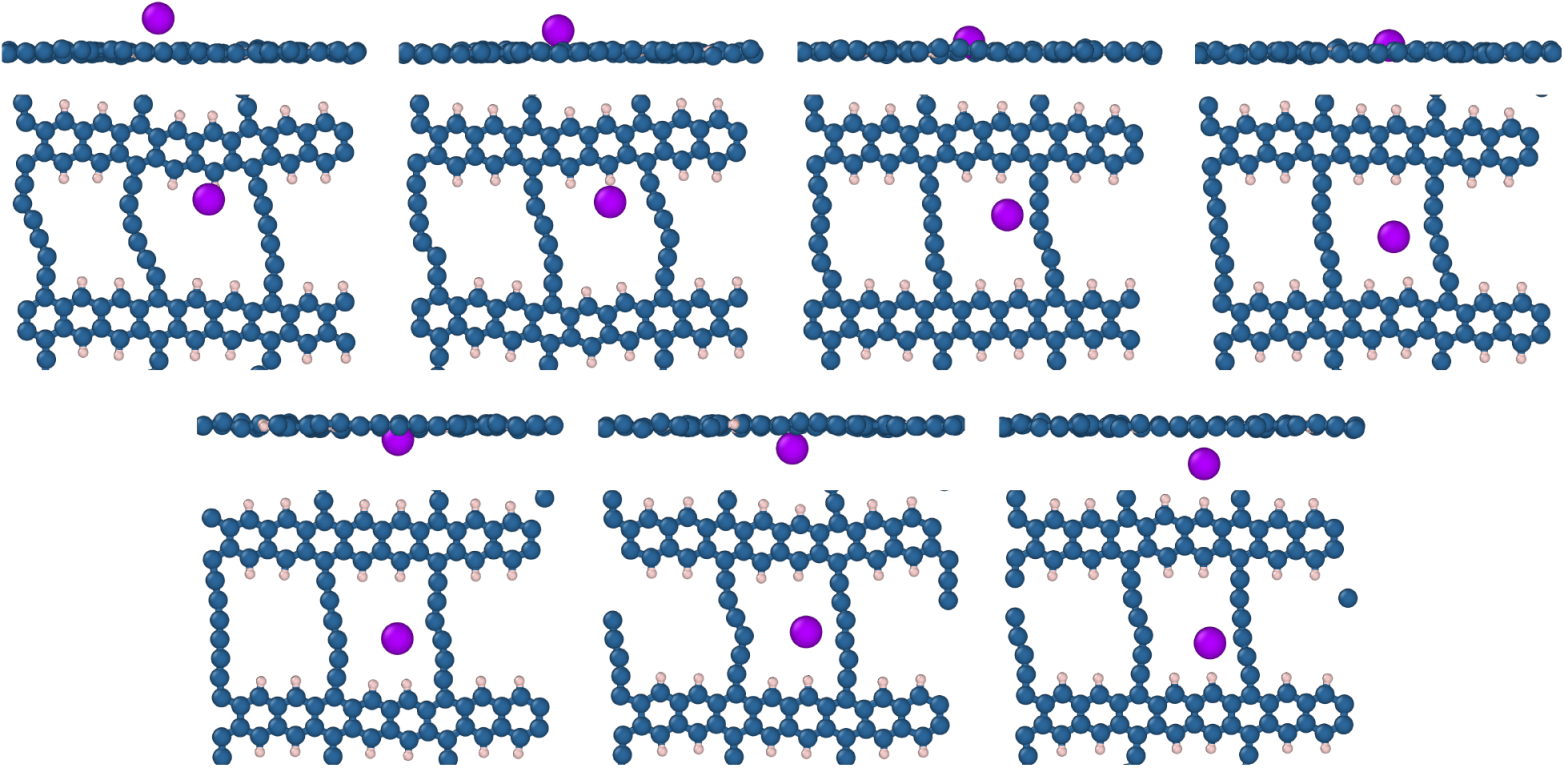
Representative snapshots, top views (upper panels) and corresponding side views (lower panels), illustrating the permeation pathway of a Na^+^ ion (purple) across [1],[3]{2}-grazyne membrane at 210 MPa. The sequence shows the ion approaching the membrane from the bulk solution, entering the pore region, and crossing the pore. The grazyne membrane is depicted with C atoms in blue and H atoms in white. Water molecules and the rest of ions have been removed for a better visualization of the process.

**Figure 12:**
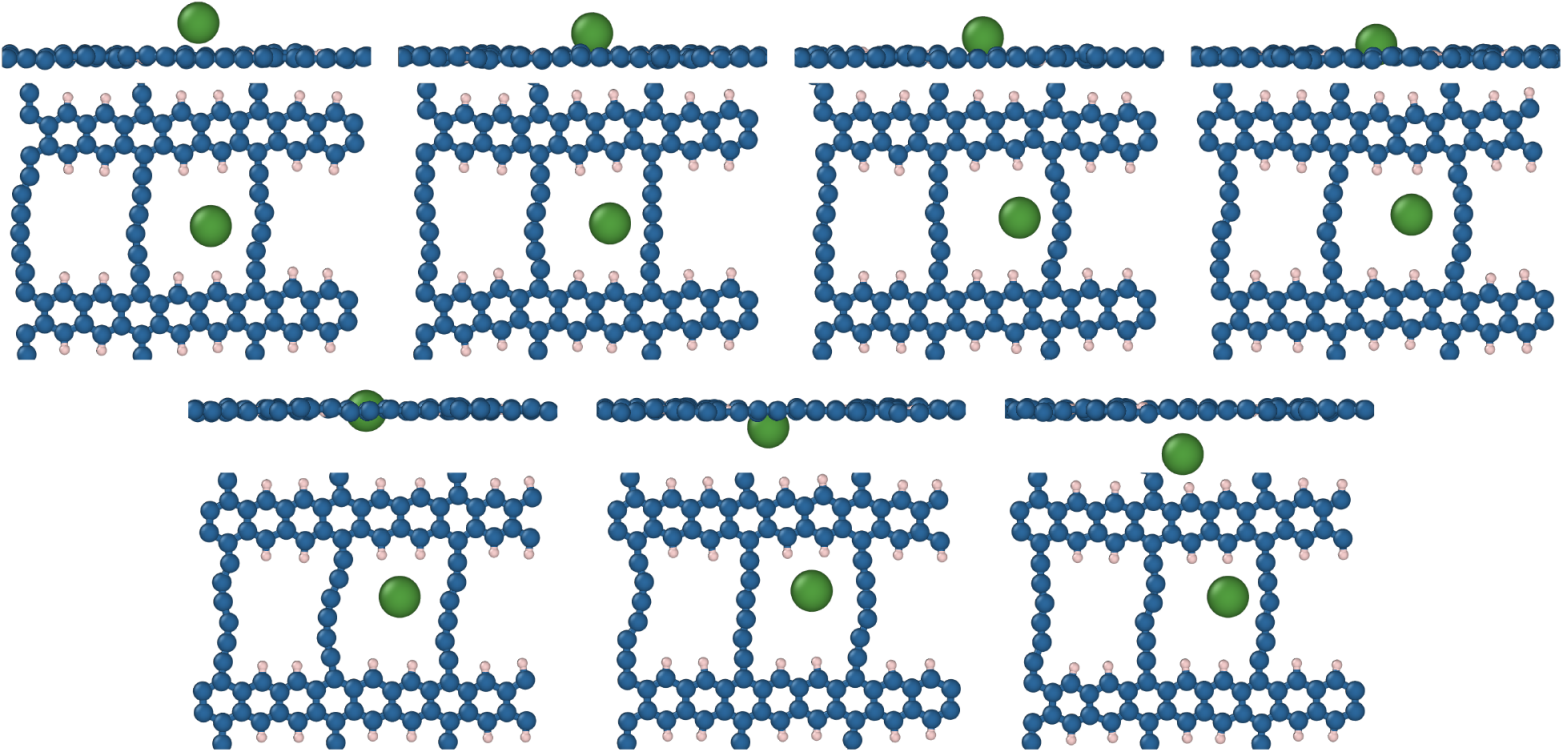
Representative snapshots, top views (upper panels) and corresponding side views (lower panels), illustrating the permeation pathway of a Cl^–^ ion (green) across [1],[3]{2}-grazyne membrane at 210 MPa. Color code as in Figure 11.

**Figure 13:**
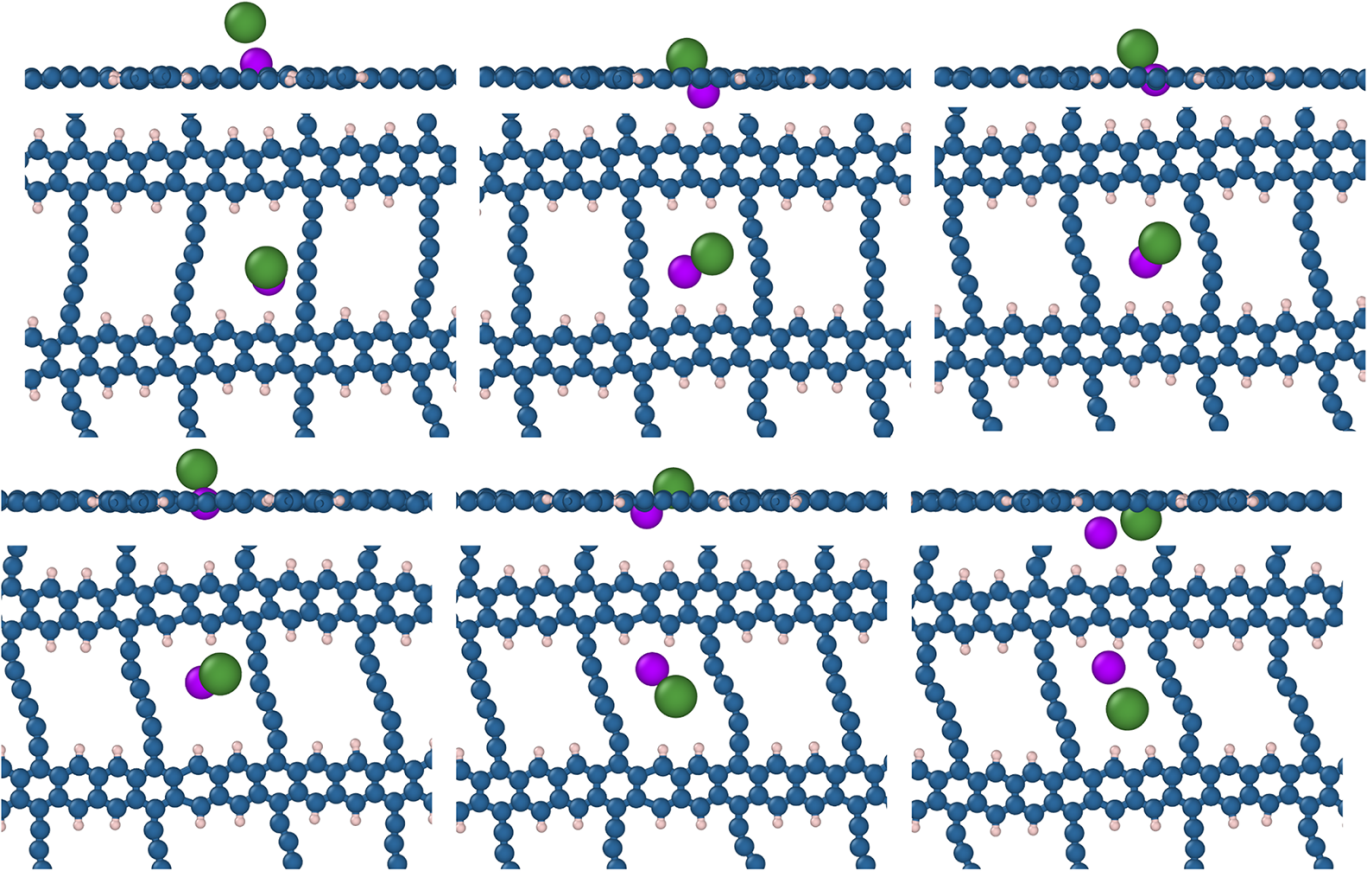
Representative snapshots, top views (upper panels) and corresponding side views (lower panels), illustrating the permeation pathway of a Cl^–^ (green) and Na^+^ (purple) pair across [1],[3]{2}-grazyne membrane at 210 MPa. Color code as in Figure 12.

## 4 Comparison with Alternative Desalination Membranes

Membrane-based technologies are increasing in importance within the field of molecular separation. In this regard, among the C-based materials studied for water desalination, nanoporous graphene stands out. For a porosity of 10%, and under conditions close to those of this work, it achieved permeabilities of up to 66 L·cm^–2^·day^–1^·MPa^–1^,^13^ with a perfect salt rejection. Studies with pristine graphynes, compounds structurally related to grazynes, demonstrated permeabilities around 13.1 L·cm^–2^·day^–1^·MPa^–1^ while maintaining perfect salt rejection.^55^ In addition, functionalized graphyne membranes were reported to reach permeabilities up to 17 L·cm^–2^·day^–1^·MPa^–1^, although in these systems the pore chemistry and local electrostatic environment are also modified by the functional groups.^56^ Clearly, grazynes fall within the same range, obtaining values of *ca.* 15 L·cm^–2^·day^–1^·MPa^–1^ when considering the best performing grazyne under conditions of perfect salt rejection. Although higher permeability values are obtained for the other grazyne structures at specific pressures, those cases are accompanied by partial ion permeation at higher applied pressures, making [1],[2]{2}-grazyne the best-balanced membrane in terms of the permeability/selectivity trade off. Studies have also been conducted with graphene hybrid membranes, such as GO-graphene systems, reaching permeabilities close to 70 L·cm^–2^·day^–1^·MPa^–1^ while maintaining full ion rejection,^57^ however, these systems involve oxidized regions and chemically heterogeneous pore environments that differ from the pristine hydrogenated grazynes considered here. Graphene foams also obtained promising results with permeabilities between 13 and 121 L·cm^–2^·day^–1^·MPa^–1^.^58^ It is important to note that this study only considered five different grazynes, with pore sizes ranging from *ca.* 1 to 21 Å^2^. Still, thanks to their high degree of modification, grazynes with larger pores and even different substituents can still be studied, allowing for better engineering, selectivity adjustment, and salt rejection. A direct comparison of these materials is summarized in Figure 14, where both permeability and ion rejection are presented. As shown, nanoporous graphene and hybrid GO–graphene membranes represent the upper limit in terms of permeability while still achieving 100% rejection. In contrast, graphyne-based systems exhibit more moderate permeabilities but maintain ideal selectivity. MoS_2_ nanopores show slightly lower permeabilities *ca.* 10 L·cm^–2^·day^–1^·MPa^–1^.^59^ It should be noted that these literature results were not obtained using the exact same parameters of the present work and some of the membranes discussed above present pore chemistries substantially different from those of the grazynes presented here, however they still provide a useful point of view. Therefore, the comparison should be interpreted qualitatively, with the aim of placing grazynes in the context of nanoporous desalination membranes. Under this perspective, the present results indicate that grazynes perform similarly to other C-based membranes while offering the additional advantage of structural tunability.

**Figure 14:**
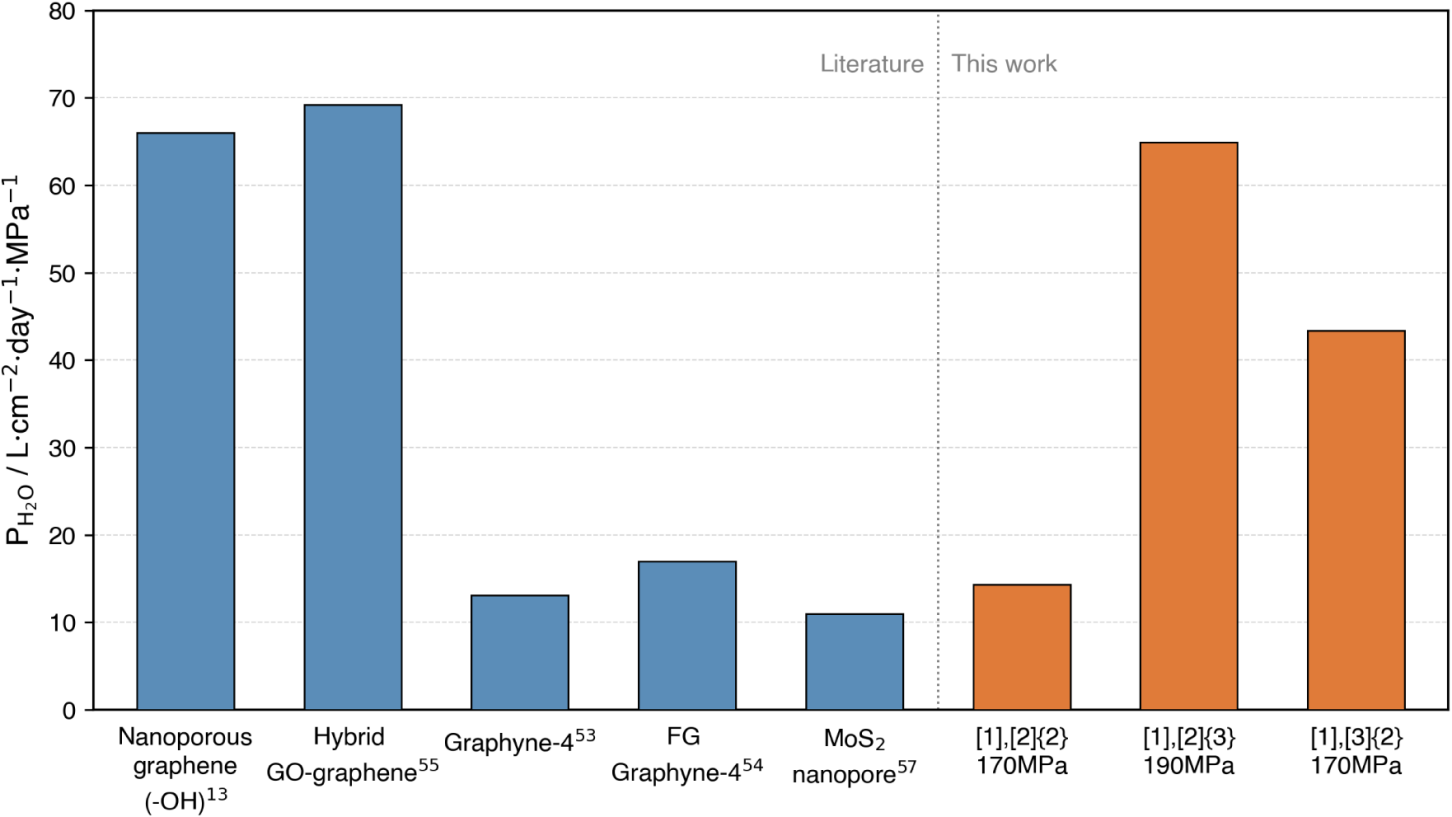
Comparison of water permeability, $P_{H_{2}O}$ , obtained in this work with representative values reported in the literature. All values correspond to a perfect ionic rejection. Blue bars represent values from the literature, while orange bars correspond to values derived from the present work.

## 5 Conclusions

The potential use of different nanostructured grazynes as desalination membranes has been explored through MD simulations. The results have shown that water transport through these membranes is highly governed by pore size and pore morphology. Grazynes with the smallest pores, named as [1],[1]{2} and [1],[2]{1}, have proven to be completely impermeable across the entire studied pressure range. Increasing the pore size, either by enlarging the acetylenic linkages or introducing vacancies to them, has consistently increased water transport. Among the three permeable membranes, a clear trend has been observed: As pressure increases, permeability increases almost linearly, consistent with transport mechanisms dominated by external forces. The grazyne with the largest pore size, the [1],[3]{2}-grazyne, has proven to be the most permeable across almost the entire range and the one with the most accessible pore for the species, as confirmed by density maps. However, this high permeability comes at the expense of allowing the passage of Na^+^ and Cl^–^ traces, thus reducing the membrane performance. On the other hand, [1],[2]{2}-grazyne has proven to be the most balanced structure, combining high water flow with almost perfect salt rejection at high pressures. This makes this grazyne the most promising candidate for the water desalination process. Finally, the [1],[2]{3}-grazyne also obtained good results, with good permeability and good salt rejection, but its water transport remained consistently lower than that of the [1],[2]{2}-grazyne. The combination of pore occupancy analysis, oxygen density maps, and PMF calculations reveals a clear pore-size dependency. Smaller pores — [1],[2]{2} and [1],[2]{3} — operate in a single-file transport, where molecules cross sequentially against a well-defined energy barrier of *ca.* 8.5 kJ·mol^–1^, while the larger pore in [1],[3]{2}-grazyne supports multimolecule transport with a lower barrier of *ca.* 5 kJ·mol^–1^. For simulations with ion flow, [1],[2]{3} obtained a TDS value of 807 mg/L. While this value is not optimal for direct consumption, it presents an EC value of *ca.* 1.21 mS·cm^–1^, which, according to FAO guidelines, places the water in the medium risk category, making it suitable for crops irrigation. However, achieving better taste levels for water remains attainable through multistage desalination cycles, taking advantage of the high flux of these membranes to meet the strictest WHO organoleptic requirements. The present work demonstrates the ability of grazynes to purify water, showing their high structural tunability and the possibility of introducing controlled modifications to their pores, representing a promising option within the family of desalination membranes. This study represents a conceptual and computational exploration of grazyne membranes at their early technological stage, laying the foundation for future experimental assessments.

## Supplementary Material



## Notes

Permeability calculation methodology; Convergence tests; radial distribution functions; coordination number and residence time analyses (PDF)
