# Supplementary material for: Molecular Dynamics Simulation of Nanostructured Grazynes for Water Desalination
Source: ACS Appl Nano Mater. 2026 Jun 29;9(27):12980–93. doi: 10.1021/acsanm.6c01675 (PMC13366747; doi:10.1021/acsanm.6c01675)
Supplement: Supplementary file 1 [file an6c01675_si_001.pdf]

Molecular Dynamics Simulation of Nanostructured Grazynes for Water Desalination

Adrià Calzada*, Francesc Viñes, and Pablo Gamallo

Departament de Ciència de Materials i Química Física & Institut de Química Teòrica i Computacional (IQTCUB), Universitat de Barcelona, c/ Martí i Franquès 1-11, Barcelona, 08028, Spain

*Corresponding author: adria.calzada@ub.edu

Section S1. Permeability Unit Conversion

To facilitate a direct comparison, the water permeability P_{H_2O} obtained from molecular dynamics simulations was converted into $L \cdot cm^{-2} \cdot day^{-1} \cdot MPa^{-1}$ using the following expression:

$$P_{H_2O} = \frac{\Delta N \cdot V_{mol}}{\Delta t \cdot A_{cell} \cdot \Delta p} \tag{1}$$

Where:

- ΔN is the net number of filtered water molecules during the steady-state period.
- V_{mol} is the volume of a single water molecule, calculated from the molar volume of water at 300 K.
- Δt is the simulation time converted to days.
- Δp is the applied pressure in MPa.
- A_{cell} is the specific area of the simulation cell.

Table S1. A_{cell} values used for [1],[2]{2}, [1],[2]{3} and [1],[3]{2}-grazynes.

Grazyne	[1],[2]{2}	[1],[2]{3}	[1],[3]{2}
A_{cell} / Å^2	853.48	1110.71	1071.63

Section S2. Validation of Simulation Time

To verify the adequacy of the 100 ps simulation time, selected simulations were extended up to 300 ps to assess the convergence of transport properties (*cf.* Figure S1). The cumulative number of permeated water molecules was then monitored as a function of time. These extended simulations show that, after the initial rapid permeation regime, the number of permeated molecules reach a clear plateau, confirming that the original simulation time is adequate for the analyzed systems.

Figure S1. Extended cumulative permeation trajectories for [1],[2]{3}-grazyne membrane after the original 100 ps diffusion window (red dashed line). Only minor additional water permeation events are observed upon extending the simulations, confirming that the original simulation time is sufficient to obtain representative transport values.

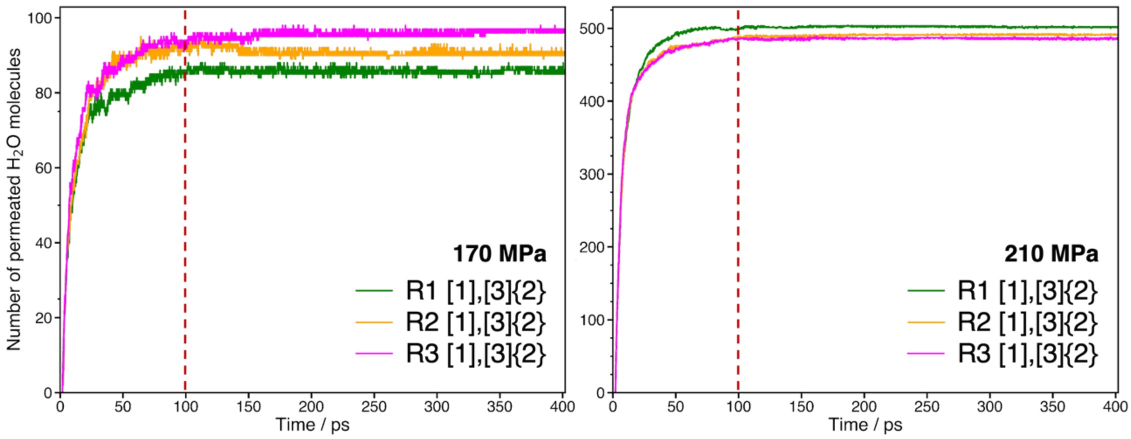

Section S3. **Radial Distribution Functions**

This section provides the normalized radial distribution functions, g(r), of the different pairs that are present in the simulations for each permeable grazyne. The RDFs confirm that the solvation structure of water and ions remains consistent across pressures and grazyne types, validating the use of the force-field parameters for all simulated conditions. Normalization was performed using the maximum value of g(r) of each dataset.

[1],[2]{2}-grazyne

Figure S2. Normalized radial distribution functions, g(r), computed in the bulk region for the [1],[2]{2}-grazyne at the three applied pressures (170, 190, and 210 MPa). The panels show the pair correlations for O_{H_2O}–O_{H_2O}, Na^+–O_{H_2O}, $Cl^- - O_{H_2O}$, Na^+–Cl^-, $Cl^- - H_{H_2O}$, and Na^+–H_{H_2O}.

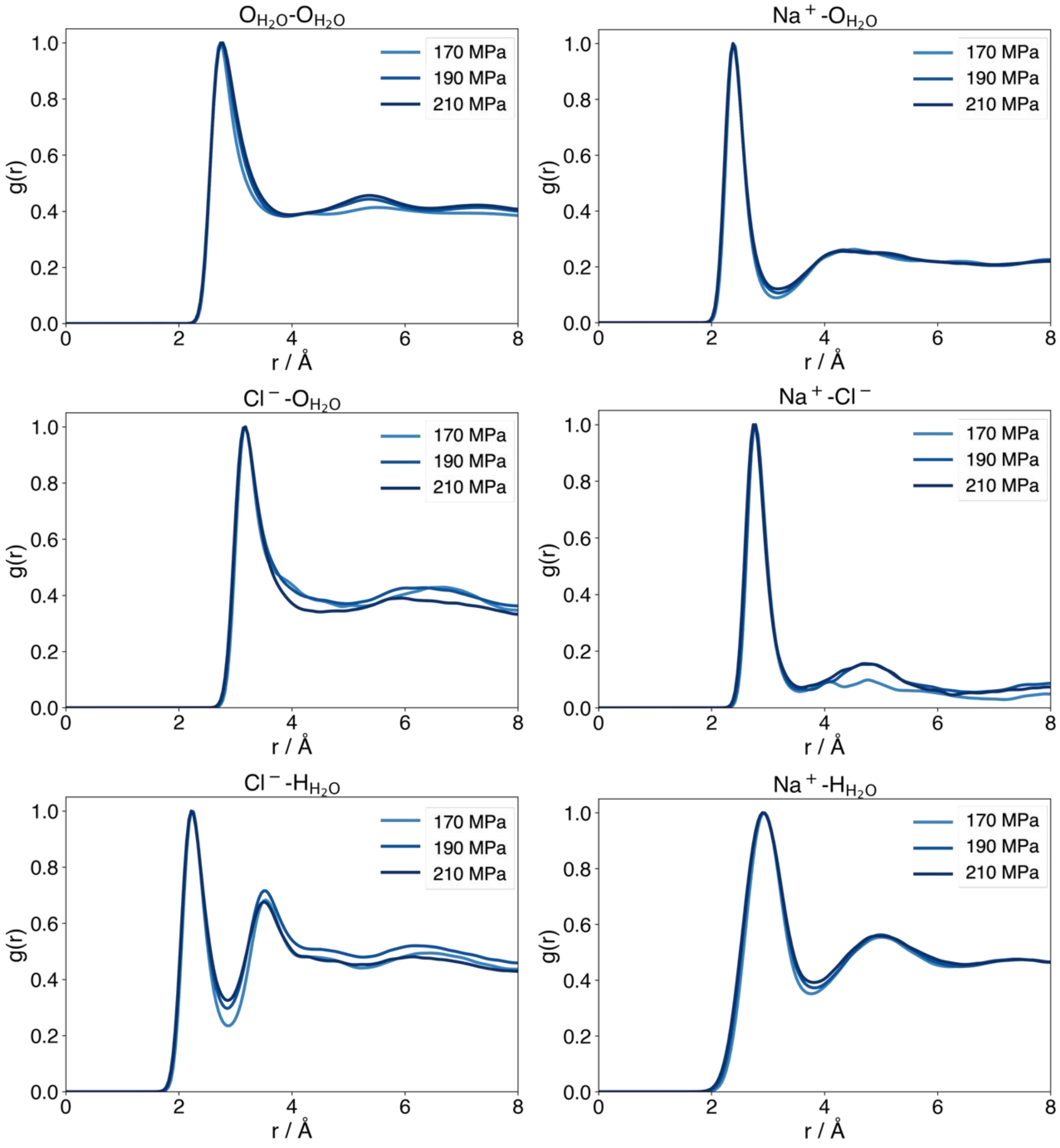

[1],[2]{2}-grazyne

Figure S3. Normalized radial distribution functions, g(r), computed in the pore region for the [1],[2]{2}-grazyne at the three applied pressures (170, 190, and 210 MPa). The panels show the pair correlations for O_{H_2O}–O_{H_2O}, Na^+–O_{H_2O}, $Cl^- - O_{H_2O}$, $Cl^- - H_{H_2O}$, and Na^+–H_{H_2O}.

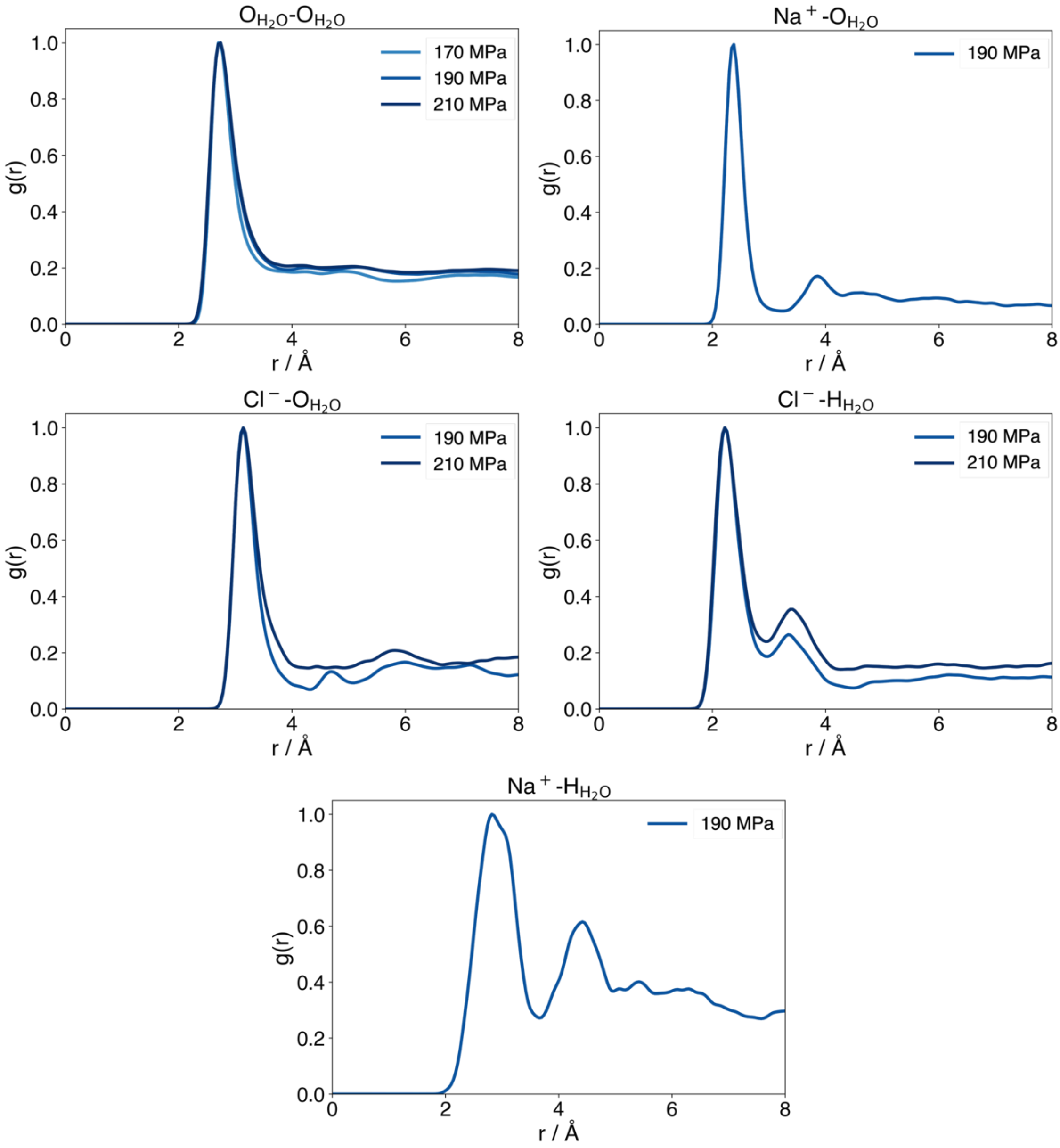

[1],[2]{3}-grazyne

Figure S4. Normalized radial distribution functions, g(r), computed in the bulk region for the [1],[2]{3}-grazyne at the three applied pressures (170, 190, and 210 MPa). The panels show the pair correlations for O_{H_2O}–O_{H_2O}, Na^+–O_{H_2O}, $Cl^- - O_{H_2O}$, Na^+–Cl^-, $Cl^- - H_{H_2O}$, and Na^+–H_{H_2O}.

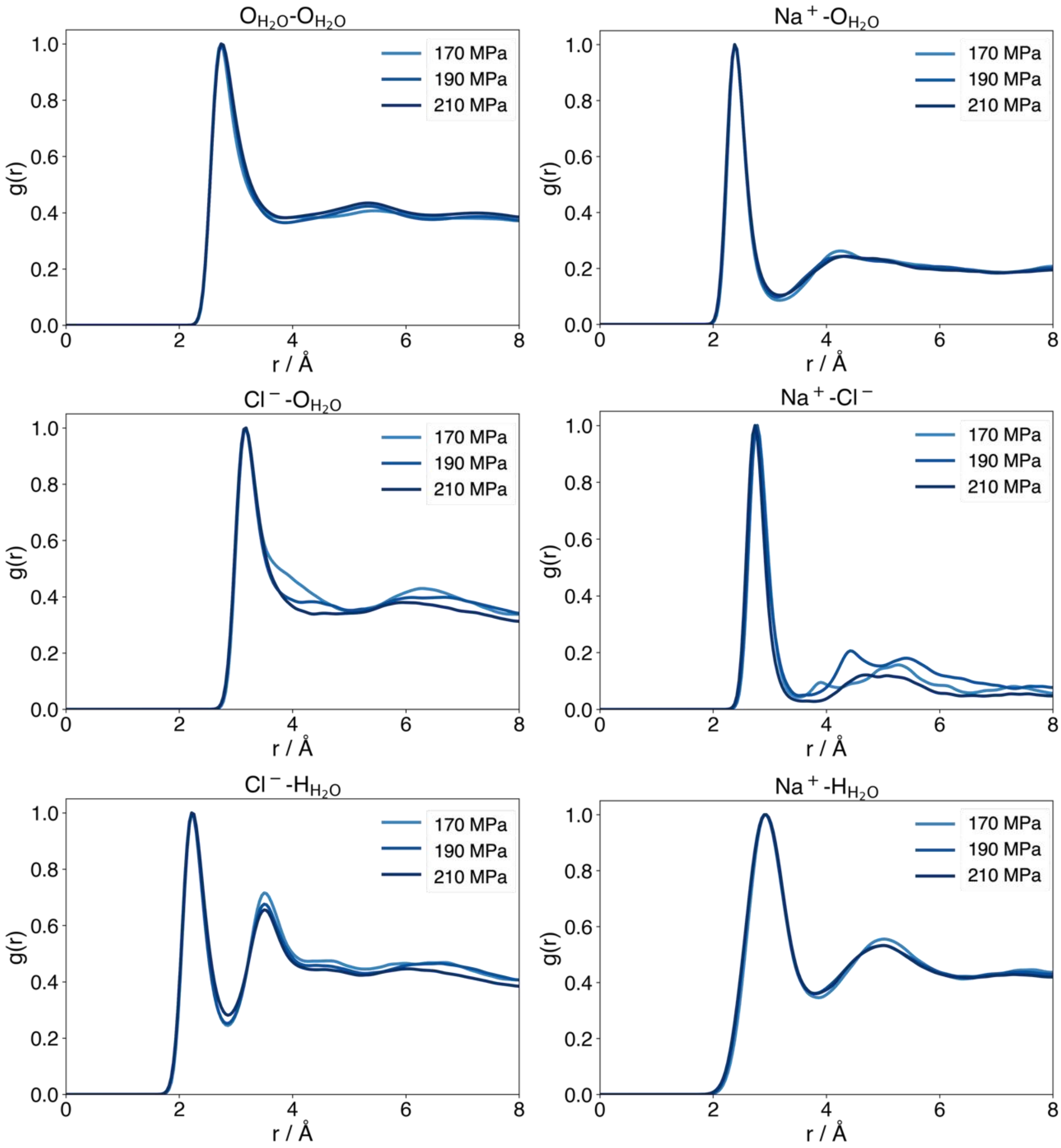

[1],[2]{3}-grazyne

Figure S5. Normalized radial distribution functions, g(r), computed in the pore region for the [1],[2]{3}-grazyne at the three applied pressures (170, 190, and 210 MPa). The panels show the pair correlations for O_{H_2O}–O_{H_2O}, Na^{+}–O_{H_2O}, $Cl^{-} - O_{H_2O}$, Na^{+}–Cl^{-}, $Cl^{-} - H_{H_2O}$, and Na^{+}–H_{H_2O}.

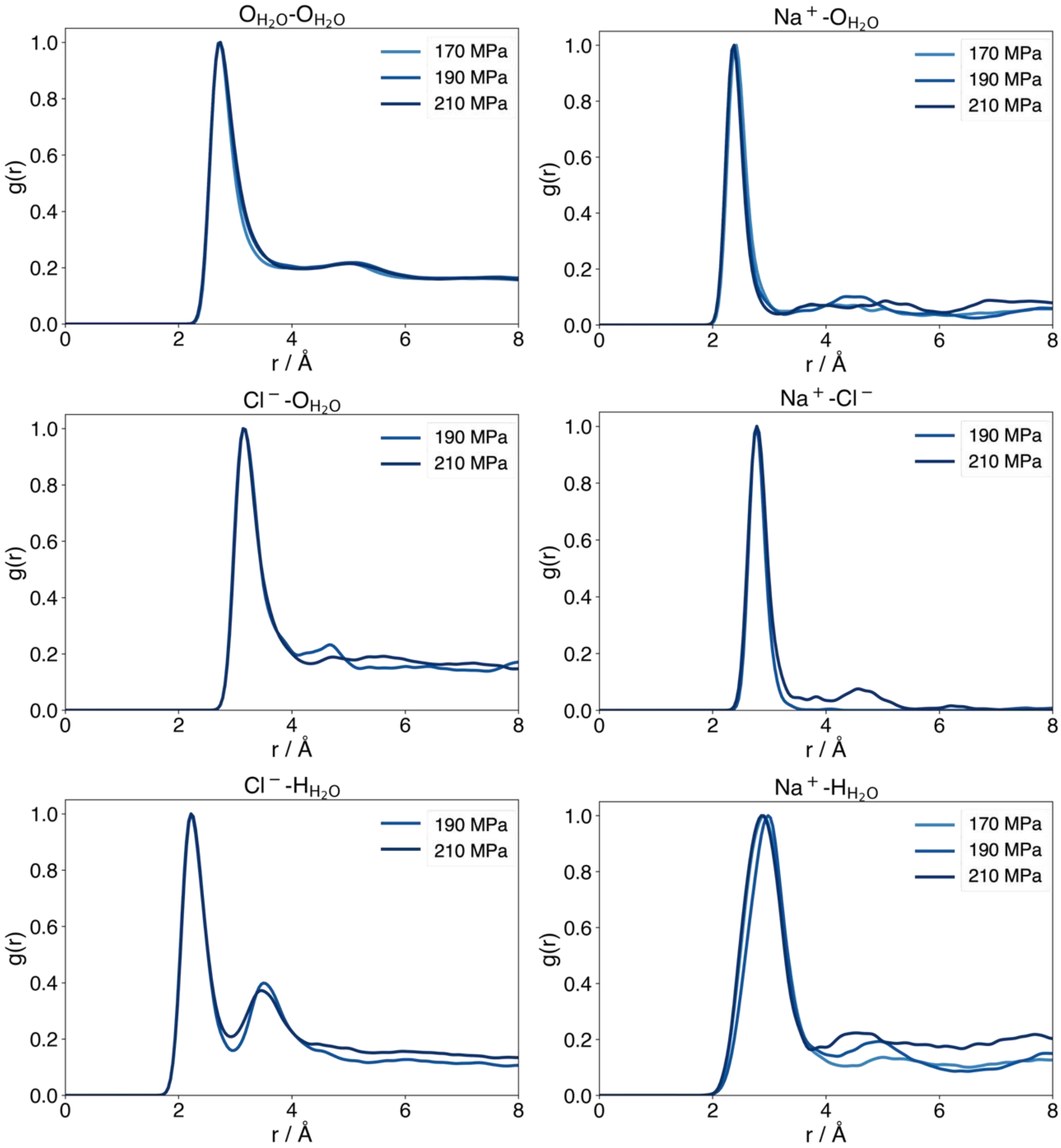

[1],[3]{2}-grazyne

Figure S6. Normalized radial distribution functions, g(r), computed in the bulk region for the [1],[3]{2}-grazyne at the three applied pressures (170, 190, and 210 MPa). The panels show the pair correlations for O_{H_2O}–O_{H_2O}, Na^+–O_{H_2O}, $Cl^- - O_{H_2O}$, Na^+–Cl^-, $Cl^- - H_{H_2O}$, and Na^+–H_{H_2O}.

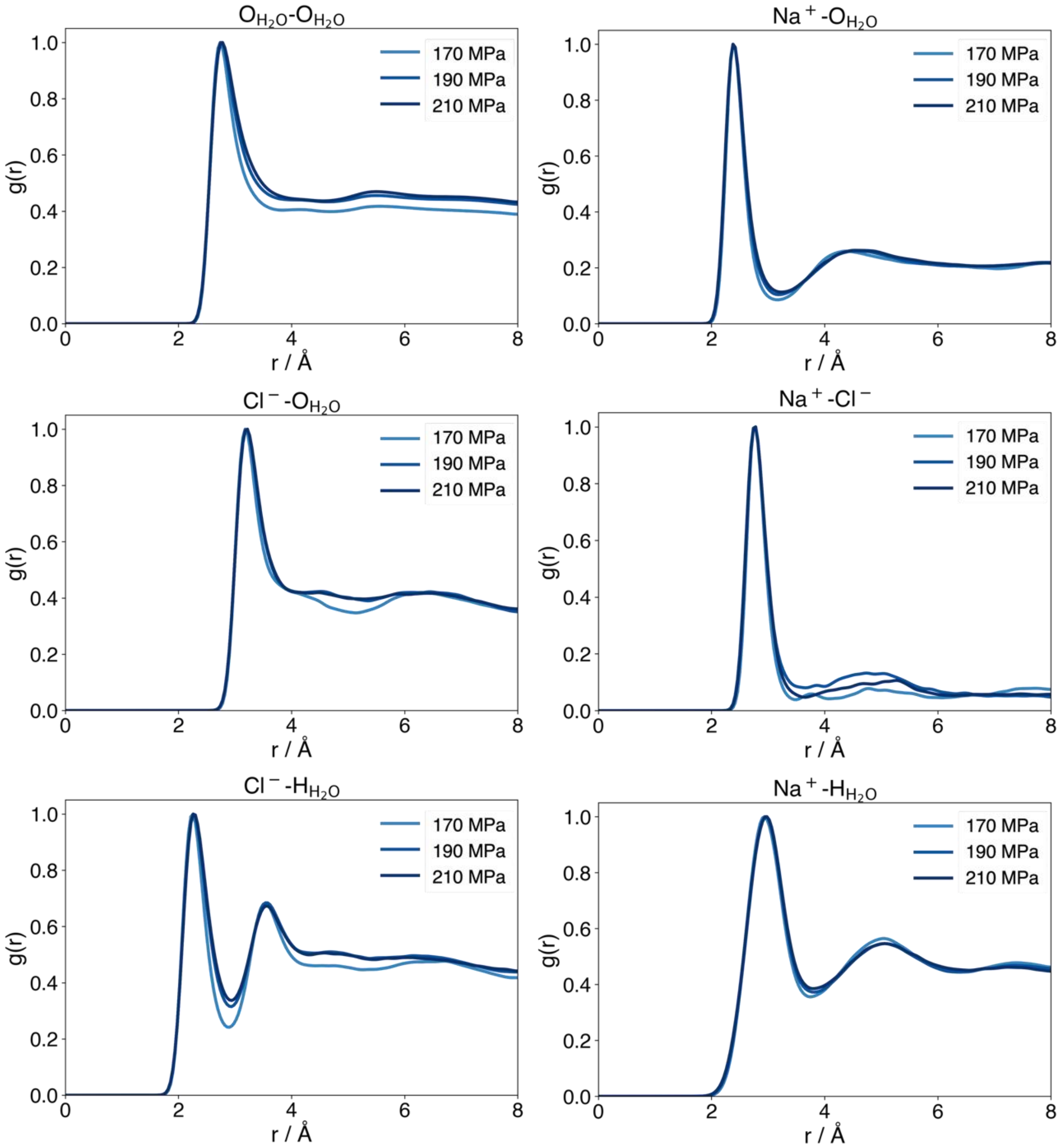

[1],[3]{2}-grazyne

Figure S7. Normalized radial distribution functions, g(r), computed in the pore region for the [1],[3]{2}-grazyne at the three applied pressures (170, 190, and 210 MPa). The panels show the pair correlations for O_{H_2O}–O_{H_2O}, Na^+–O_{H_2O}, $Cl^- - O_{H_2O}$, Na^+–Cl^-, $Cl^- - H_{H_2O}$, and Na^+–H_{H_2O}.

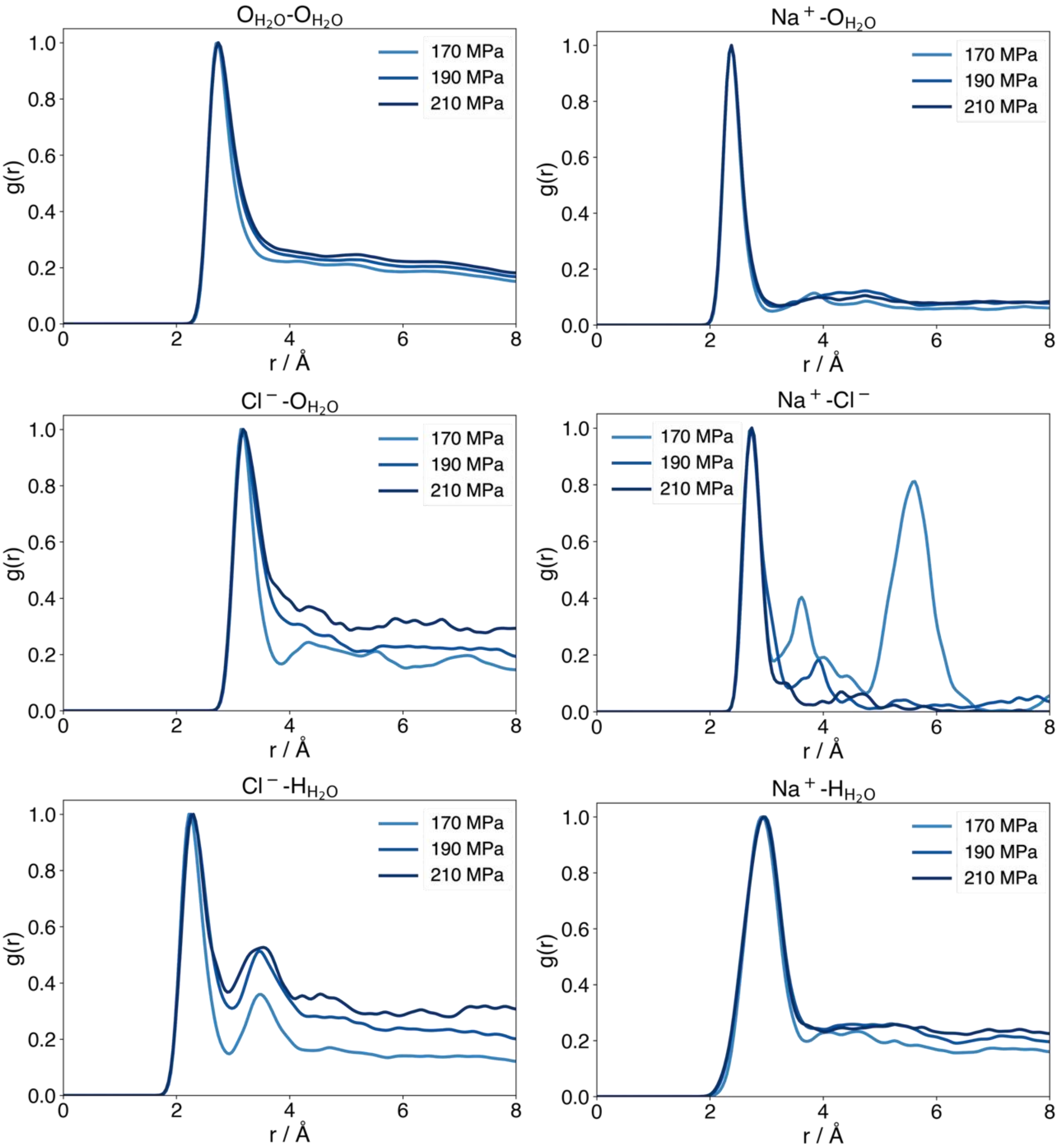

Section S4. Coordination Number and Residence Times

This section provides the coordination number, CN, and residence times, τ, computed for both Na^+ and Cl- for each permeable grazyne, considering $Na^+–O_{H_2O}$ and $Cl^-–H_{H_2O}$, respectively.

Table S2. Coordination number, CN, and residence times, τ, computed in the pore and bulk region for the [1],[2]{2}, [1],[2]{3}, and [1],[3]{2}-grazynes at the three permeable applied pressures (170, 190, and 210 MPa).

Grazyne	p / MPa	CN Na^+		CN Cl^-		τ Na^+ / ps		τ Cl^- / ps	
		Bulk	Pore	Bulk	Pore	Bulk	Pore	Bulk	Pore
[1],[2]{2}	170	5.80	—	6.12	—	55.8	—	39.9	—
	190	6.09	4.64	6.33	5.30	25.7	18.4	21.6	30.7
	210	6.04	5.41	6.62	5.90	16.4	16.5	16.0	12.2
[1],[2]{3}	170	6.01	5.39	5.95	—	57.5	39.7	35.9	—
	190	5.84	6.10	6.27	5.97	35.7	25.1	29.2	30.6
	210	6.02	5.00	6.41	5.44	22.2	29.9	18.8	24.0
[1],[3]{2}	170	5.99	5.13	6.30	5.32	41.2	23.3	32.3	38.3
	190	5.50	4.74	5.92	5.04	12.6	12.2	9.9	4.5
	210	5.46	4.71	5.85	4.50	9.0	7.2	7.5	4.9
